# Herbs and Spices Modulate Gut Bacterial Composition in Adults at Risk for CVD: Results of a Prespecified Exploratory Analysis from a Randomized, Crossover, Controlled-Feeding Study

**DOI:** 10.1093/jn/nxac201

**Published:** 2022-09-02

**Authors:** Kristina S Petersen, Samantha Anderson, Jeremy R Chen See, Jillian Leister, Penny M Kris-Etherton, Regina Lamendella

**Affiliations:** Department of Nutritional Sciences, Texas Tech University, Lubbock, TX, USA; Department of Nutritional Sciences, The Pennsylvania State University, University Park, PA, USA; Department of Biology, Juniata College, Huntingdon, PA, USA; Wright Labs, LLC, Huntingdon, PA, USA; Department of Biology, Juniata College, Huntingdon, PA, USA; Wright Labs, LLC, Huntingdon, PA, USA; Department of Biology, Juniata College, Huntingdon, PA, USA; Wright Labs, LLC, Huntingdon, PA, USA; Department of Nutritional Sciences, The Pennsylvania State University, University Park, PA, USA; Department of Biology, Juniata College, Huntingdon, PA, USA; Wright Labs, LLC, Huntingdon, PA, USA

**Keywords:** herbs, spices, polyphenols, microbiota, microbiome, bacteria

## Abstract

**Background:**

Herbs and spices are rich in polyphenolic compounds that may influence gut bacterial composition. The effect of culinary doses of herbs and spices consumed as part of a well-defined dietary pattern on gut bacterial composition has not been previously studied.

**Objectives:**

The aim of this prespecified exploratory analysis was to examine gut bacterial composition following an average American diet (carbohydrate: 50% kcal; protein: 17%; total fat: 33%; saturated fat: 11%) containing herbs and spices at 0.5, 3.3, and 6.6 g.d^–1^.2100 kcal^–1^ [low-, moderate-, and high-spice diets, respectively (LSD, MSD, and HSD)] in adults at risk for CVD.

**Methods:**

Fifty-four adults (57% female; mean ± SD age: 45 ± 11 y; BMI: 29.8 ± 2.9 kg/m^2^; waist circumference: 102.8 ± 7.1 cm) were included in this 3-period, randomized, crossover, controlled-feeding study. Each diet was provided for 4 wk with a minimum 2-wk washout period. At baseline and the end of each diet period, participants provided a fecal sample for 16S rRNA gene (V4 region) sequencing. QIIME2 was used for data filtration, sequence clustering, taxonomy assignment, and statistical analysis.

**Results:**

α-diversity assessed by the observed features metric ( *P* = 0.046) was significantly greater following the MSD as compared with the LSD; no other between-diet differences in α-diversity were detected. Differences in β-diversity were not observed between the diets ( *P* = 0.45). Compared with baseline, β-diversity differed following all diets ( *P* < .02). Enrichment of the Ruminococcaceae family was observed following the HSD as compared with the MSD (relative abundance = 22.14%, linear discriminant analysis = 4.22, *P* = 0.03) and the LSD (relative abundance  = 24.90%, linear discriminant analysis = 4.47, *P* = 0.004).

**Conclusions:**

The addition of herbs and spices to an average American diet induced shifts in gut bacterial composition after 4 wk in adults at risk for CVD. The metabolic implications of these changes merit further investigation. This trial was registered at clinicaltrials.gov as NCT03064932.

## Introduction

Diet, especially nondigestible dietary compounds, shapes gut bacterial composition by serving as the substrates for bacterial metabolism. Evaluation of fecal bacterial changes in response to dietary intervention reveals signatures reflective of intake patterns. Shinn et al. (1) recently showed dietary intake of specific whole foods (almonds, avocados, broccoli, walnuts, whole grain barley, and whole grain oats) could be predicted with 70%–85% accuracy from the relative abundance of 15–22 fecal bacteria. These foods are all sources of fiber, a well-established modulator of gut bacterial composition (2).

Polyphenols—a large class of structurally diverse chemicals, including phenolic acids (derivatives of benzoic acid and cinnamic acid) and stilbenoids (stilbenes)—are usually poorly absorbed in the upper gastrointestinal tract and pass into the large intestine, where they become accessible to gut bacteria. In vivo studies show that gut bacteria have a strong influence on the bioavailability and bioactivity of a range of dietary polyphenols (2). Herbs and spices are rich in polyphenolic compounds (3, 4) that may influence bacterial composition. An analysis of herbs and spices from the botanical families Lamiaceae (rosemary, thyme, and oregano), Apiaceae (cumin), and Lauraceae (cinnamon and bay leaf) identified 52 phenolic compounds with clear differences in composition by family (4). However, to date, there has been limited investigation of the effect of herbs and spices on gut bacterial composition. A randomized, placebo-controlled, double-blinded pilot study showed that intake of a 5-g capsule containing spices [cinnamon: 1 g (20%); oregano: 1.5 g (30%); ginger: 1.5 g (30%); black pepper: 0.85 g (17%); cayenne pepper: 0.15 g (3%)] resulted in a difference in 26 operational taxonomic units (OTUs) when compared with a placebo (maltodextrin) after 2 wk (5).

This suggests that herbs and spices and their phenolic constituents serve as substrates for gut bacteria and induce compositional changes; however, this was a relatively short study, and the herbs and spices were given in capsule form, which is not representative of culinary exposure. Furthermore, the results are potentially confounded by the background diet. To our knowledge, no randomized controlled trials have examined the effect of repeated exposure (longer-term intake) to culinary doses of mixed herbs and spices as part of well-defined dietary patterns on gut bacterial composition.

The present prespecified exploratory analysis examined the effect of an average American diet (carbohydrate: 50% kcal; protein: 17%; total fat: 33%; saturated fat: 11%; sodium: 3000 mg/d; fiber: 22 g/d) containing herbs and spices at 0.5, 3.3, and 6.6 g.d^–1^.2100 kcal^–1^ [low-, moderate-, and high-spice diets, respectively (LSD, MSD, and HSD)] on gut bacterial composition in adults at risk for CVD. It was hypothesized that herbs and spices would affect gut bacterial composition in a dose–response manner in adults at risk for CVD, which is a population where aberrant microbiota may contribute to the development of CVD and other metabolic diseases (6).

## Methods

### Study design

Details of the study design and primary and secondary outcomes are reported elsewhere (7, 8). Data are reported here for compositional changes in the gut bacteria, a prespecified exploratory outcome. Briefly, a 3-period, randomized, crossover, controlled-feeding study was conducted to examine the dose–response effects of including herbs and spices in a diet approximately representing average American macronutrient intake (9) (carbohydrate: 50% kcal; protein: 17%; total fat: 33%; saturated fat: 11%; sodium: 3000 mg/d; fiber: 22 g/d). Complete diets were provided to the participants that had the following quantities of dried herbs and spices (incorporated on a grams-per-kilocalorie basis into recipes): *1*) low dose, 0.5 g.d^–1^.2100 kcal^–1^ (LSD); *2*) moderate dose, 3.3 g.d^–1^.2100 kcal^–1^ (MSD); and *3*) high dose, 6.6 g.d^–1^.2100 kcal^–1^ (HSD). **Supplemental Tables 1–3** show the herb/spice composition of the diets, the nutrient composition of the background diet, and the 7-d menu used in the study. Each diet was consumed for 4 wk with a minimum 2-wk washout period (median break: 19 d; range: 14–69 d). Participants who consented to participate in this substudy provided fecal samples at baseline and the end of each diet period for these analyses. A computer-generated 6-sequence scheme (randomization.com) that contained blocks of 6 sequences was used for randomization. The Institutional Review Board at Pennsylvania State University approved the protocol, and all participants gave informed consent. The trial is registered at clinicaltrials.gov (NCT03064932).

### Participants

Participants were recruited from the State College, PA, area. Eligible individuals were aged 30 to 75 y and had a BMI ≥25 to ≤35 kg/m^2^, abdominal obesity (men, ≥94 cm; women, ≥80 cm), and at least 1 other risk factor for CVD. CVD risk factors were defined as follows: *1*) elevated glucose (≥100 and ≤126 mg/dL), *2*) low HDL cholesterol (men, <40 mg/dL; women, <50 mg/dL), *3*) elevated triglycerides (≥150 mg/dL and ≤300 mg/dL), *4*) high blood pressure (≥130/85 and ≤160/100 mm Hg), *5*) elevated LDL cholesterol (>130 mg/dL), and *6*) elevated high-sensitivity CRP (hs-CRP; >1 mg/L). Exclusion criteria were as follows: current or recent (≤6 mo) use of tobacco products; >10% change in body weight in the previous 6 mo; use of medications or over-the-counter products that lower blood pressure, cholesterol, or glucose; oral steroids; consumption of >14 alcoholic beverages per week; CVD, type 1 or 2 diabetes, liver disease, cancer, or inflammatory conditions (e.g., gastrointestinal disorders, rheumatoid arthritis); pregnancy or breastfeeding within the previous 12 mo; and allergies, intolerance, or aversions to foods included in the study menu.

### Baseline cardiovascular risk factor assessment

The methods used for assessment of CVD risk factors have been published (7). Briefly, baseline testing was conducted on 2 separate days following a 12-h fast and avoidance of alcohol and over-the-counter medication for 48 h. On both days, weight was measured, and a fasting blood draw was taken for analysis of lipids and lipoproteins, glucose, and hs-CRP. On one of the test days, blood pressure and waist circumference were measured. Waist circumference was measured at the iliac crest by 2 nurses while participants were standing with their feet shoulder-width apart with clothing removed from the waistline. Two measurements were taken to 0.1 cm and averaged; if measurements differed by >0.5 cm, a third measurement was taken, and the 2 closest measures were averaged.

Plasma glucose and serum total cholesterol, LDL cholesterol, HDL cholesterol, triglycerides, and hs-CRP were measured in samples from both test days by the Pennsylvania State University Biomarker Core Lab using a Cobas c311 chemistry analyzer (Roche Diagnostics). Day 1 and 2 values were averaged for data analysis. Blood pressure was measured with an automated sphygmomanometer (SphygmoCor XCEL; AtCor Medical). Measurements were performed in the seated position after a 5-min rest period; 3 measurements were taken, and the average of the last 2 measurements was used for data analysis. If systolic blood pressure was inconsistent (i.e., difference >10 mm Hg), a fourth measurement was taken, and the 2 closest measurements were averaged.

### Fecal sample collection

Participants collected 1 fecal sample from a single defecation at baseline and the end of each diet period using a provided collection kit (Ziploc bags, cooler, ice pack, nonlatex gloves, a long-handled spoon, a stool collection hat, and 2- to 30-mL Para-Pak Clean Vials; Meridian Bioscience). Participants were instructed to store the fecal sample in the freezer until it was delivered in a cooler with an ice pack to the clinical research center. Samples were stored at –80 °C until analysis.

### DNA extraction and quantification

DNA was extracted from samples using the ZymoBIOMICS DNA/RNA Miniprep Kit (Zymo Research) according to the manufacturer's protocol and eluted using 50 μL of DNase/RNase–free water. After extraction, samples were quantified with a Qubit 4 Fluorometer and 1X Qubit dsDNA High Sensitivity Assay Kit (Thermo Fisher Scientific).

### PCR amplification

All 16S rRNA Illumina-tag PCR reactions were performed on DNA extracts of the V4 hypervariable region per the Earth Microbiome Project's protocol (10). PCR products were pooled equimolarly and gel purified on a 2% agarose gel via the QIAquick Gel Purification Kit (Qiagen). Before sequencing, the purified pool was quality checked with an Agilent 2100 BioAnalyzer and Agilent DNA High Sensitivity DNA Kit (Agilent Technologies). The purified pool was stored at –20 °C and then sequenced by Wright Labs LLC using an Illumina MiSeq v2 chemistry kit with paired-end 250-bp reads.

### Quality filtering and amplicon sequence variant picking

Raw data were imported into QIIME2 for processing and analyses (11). Initial-quality Phred *Q* scores were determined using QIIME2, while the cumulative expected error for each position was determined with VSEARCH (12). Based on these quality data, forward reads were truncated at a length of 241, with a maximum expected error of 0.5, and reverse reads were truncated at a length of 198, with a maximum expected error of 0.5 within QIIME2’s implementation of the DADA2 pipeline (13). QIIME2’s DADA2 pipeline was also used to merge forward and reverse reads, remove chimeras, and assign the remaining sequences to amplicon sequence variants (ASVs).

Representative sequences were used to determine taxonomic information for the ASVs using a naive Bayes classifier as implemented in QIIME2’s “qiime feature-classifier classify-sklearn” command, with a pretrained Silva 132 database containing 515F/806R sequences (14). Representative sequences were also used to create a rooted phylogenetic tree using MAFFT (15) and FastTree2 (16) through QIIME2’s “qiime phylogeny align-to-tree-mafft-fasttree” command.

ASVs identified as mitochondria or chloroplasts were removed since these likely represent eukaryotic contamination instead of true bacterial signal. Samples with <1000 sequences remaining after filtration were removed from the ASV table.

### Statistical analyses

#### α-diversity analysis

α-diversity was calculated by subsampling the ASV table at 10 depths, ranging from 800 to 8000 sequences, for the following metrics: Faith's phylogenetic diversity (17), observed features (11), and Pielou's evenness (18). At each depth, 20 iterations were performed to obtain mean α-diversity values for the different metrics. A rarefaction plot was created with the results of this subsampling to confirm that diversity approached an asymptote and slope decreased as depth increased. The mixed models procedure (PROC MIXED, SAS version 9.4; SAS Institute) was used to examine the effect of diet on each aforementioned α-diversity metric. Participant was modeled as a repeated effect to account for the repeated measures crossover design. Diet was modeled as a fixed effect. Diet period was included as a fixed effect, and the diet period × diet interaction was examined for evidence of carryover effects. No significant diet period × diet interactions were observed, so diet period was removed from the final model. When a main diet effect was detected, post hoc tests were conducted and adjusted for multiple comparisons using the Tukey-Kramer method. The normality of the residuals was assessed using univariate analysis (PROC UNIVARIATE) to quantitatively evaluate skewness and to visually inspect the distribution and normal probability (*Q*-*Q*) plots. Selection of model covariance structures was based on optimizing fit statistics (evaluated as the lowest Bayesian information criterion). In the primary analyses, the between-diet difference was assessed. Secondary analyses examined the within-diet change in each α-diversity metric relative to baseline (PROC MIXED).

#### β-diversity analysis

β-diversity analyses were conducted after the ASV table had first undergone cumulative sum scaling normalization (19) to mitigate differences between samples attributed to sequencing depth. Distances between samples were calculated using the weighted UniFrac metric (20) based on the normalized table and rooted tree. The resulting distance matrix was visualized by a principal coordinates analysis plot. Statistical differences between sample groupings based on diet were evaluated by ANOVA using distance matrices with permutations constrained by individual (Adonis 2, *P* ≤ 0.05). Statistical differences between sample groupings based on diet period and the diet × diet period interaction were also examined to determine the potential for carryover effect; no statistically significant effects were observed (data not presented).

#### Taxonomic comparisons

Biomarker analysis was performed using linear discriminant analysis effect size (LEfSe) (21) to identify pairwise differences in enriched taxa between the diets and between baseline and each diet. The ASV table was collapsed to level 7 (species) and normalized with the CPM method (counts per million), in which raw counts were divided by the sum of the counts per sample and the resulting dividend was multiplied by 1 million. Linear discriminant analysis (LDA) was used to estimate the effect size of each feature using LEfSe, with an α level of 1.0 to disable the Kruskal-Wallis test *P* value filtering, since the data are not from independent groups (test assumption). Features with a log(LDA) score ≥2.0 were then tested for statistical significance with Wilcoxon signed rank tests (rstatix package, R; R Foundation for Statistical Computing) to account for the paired nature of the data. Features that yielded a Wilcoxon signed rank *P* value ≤0.05 and a log(LDA) score ≥2.0 were considered to be differential. LEfSe was used because this method emphasizes statistical significance, biological consistency, and effect relevance, therefore enabling identification of differentially abundant features consistent with biologically meaningful categories (21).

#### Predictive functional analysis

Phylogenetic Investigation of Communities by Reconstruction of Unobserved States 2 (PICRUSt2) was used to predict the functional capabilities of the changes in gut bacteria composition (22). Predicted KEGG orthologs (Kyoto Encyclopedia of Genes and Genomes) were regrouped into KEGG pathways using a custom Python script. Both predictive functional data sets were then CPM normalized (counts per million). LEfSe was used with the normalized data for between- and within-diet change from baseline comparisons, with the α level set to 1.0 to disable the Kruskal-Wallis *P* value filtering as previously described. Only features identified as having significantly differential abundance (Wilcoxon signed rank test, *P* ≤ 0.05) with a log(LDA) score of at least 2.0 were considered to be enriched.

## Results

### Participants

Of the 71 participants randomized, 56 consented to participate in the microbiota substudy. In total, 54 participants provided at least 1 sample; 1 participant withdrew during baseline testing and 1 subsequently declined to provide samples. At baseline, all participants provided a fecal sample; however, 2 samples were excluded from data analysis because <1000 sequences remained after quality filtration. In total, data analyses included samples from 44, 52, and 47 participants following the LSD, MSD, and HSD, respectively (**Supplemental Figure 1**). On ≥93% of study days, participants self-reported consuming all of the provided foods.

**Table 1** presents the baseline characteristics of the 54 participants included in the data analyses. The cohort was 57% female and had a mean ± SD age of 45 ± 11 y, BMI of 29.8 ± 2.9, and waist circumference of 102.8 ± 7.1 cm. The baseline characteristics of the participants in this substudy are comparable to the baseline characteristics of the entire cohort (**Supplemental Table 4**).

**TABLE 1:** Baseline characteristics of the analytic sample including participants at risk of CVD^1^

Characteristic	Value
Sex	
Male	23 (43)
Female	31 (57)
Age, y	45 ± 11
BMI, kg/m^2^	29.8 ± 2.9
Waist circumference, cm	102.8 ± 7.1
Male	103.2 ± 6.0
Female	102.5 ± 7.8
Cholesterol, mg/dL	
Total	193 ± 33
LDL	126 ± 28
HDL	49 ± 11
Triglycerides, mg/dL	105 [79–117]
Glucose,^2^ mg/dL	99 ± 7
High-sensitivity CRP, ^3^ mg/L	2.5 [1.0–4.6]
Blood pressure, mm Hg	
Systolic	129 ± 13
Diastolic	81 ± 10

1 Values are *n* (%), mean ± SD, or median [IQR]. *n* = 54. All characteristics were measured in the fasting state. Biological analytes were measured in serum unless otherwise stated. CRP, C-reactive protein; CVD, cardiovascular disease.

2 Measured in plasma.

3 Values >10 mg/L were excluded from analysis because this is indicative of acute illness; *n* = 52.

### PCR amplification, sequencing, and ASV assignment

16S rRNA gene PCR amplification of the V4 region was completed for all samples. High-quality sequence data were obtained for 195 of 202 fecal samples (**Supplemental Table 5**). Overall, sequencing depth ranged from 2937 to 276,710 sequences per sample. A total of 5,578,974 sequences were obtained after quality filtering and merging, as well as chimera, chloroplast, and mitochondria removal. After quality filtering, sequence counts ranged from 1933 to 130,595 sequences per sample for analysis. An overall 195 samples had a sequencing depth exceeding 1000 sequences and were incorporated into the community analyses and a cumulative sum scaling–normalized ASV table.

### α- and β-diversity

**FIGURE 1:**
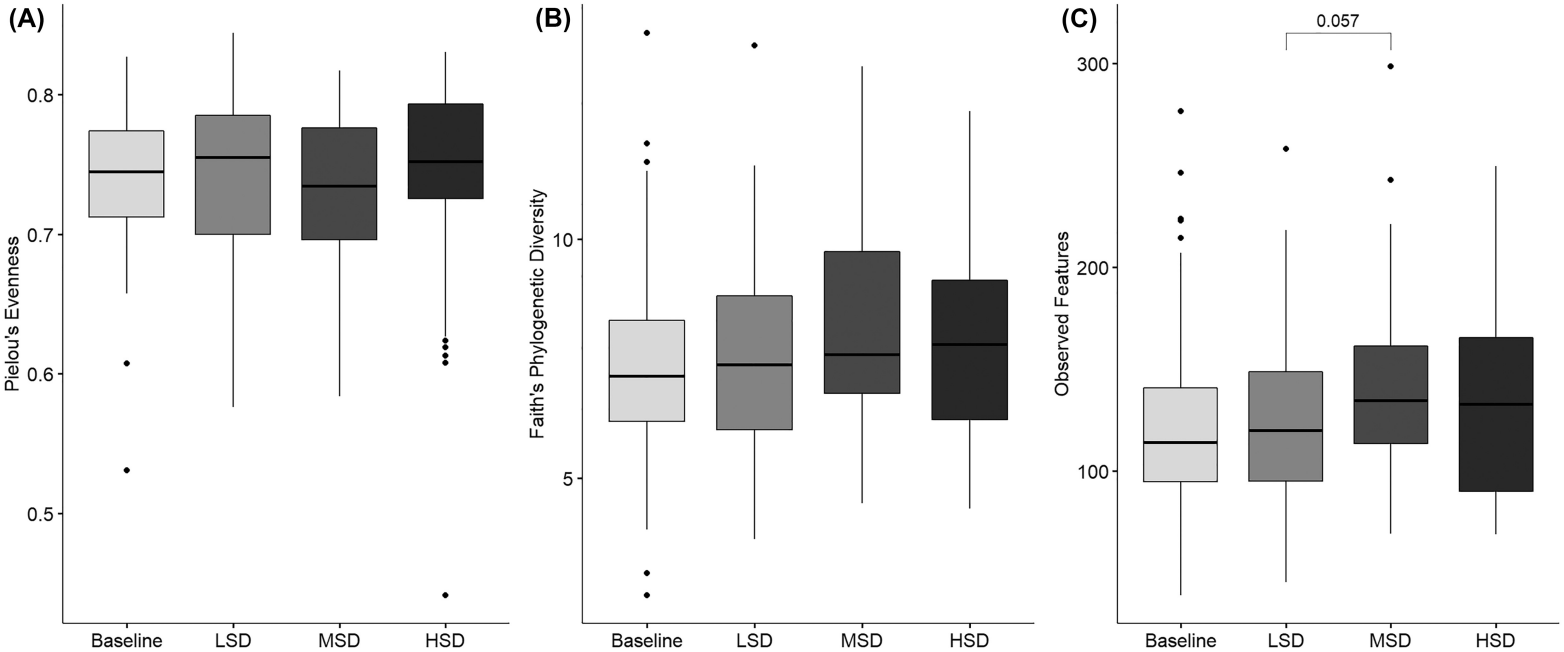
α-diversity values at baseline and following each spice-containing diet based on the following metrics: (A) Pielou's evenness, (B) Faith's phylogenetic diversity, and (C) observed features. Values are presented as median (line), IQR (box), 95% CI (error bars), and outliers (circles). Statistical analyses were performed with SAS version 9.4 (SAS Institute). The mixed models procedure (PROC MIXED) was used to determine the effect of diet on each α-diversity metric. When a main effect was detected, post hoc tests were conducted and adjusted for multiple comparisons using the Tukey–Kramer method. Tukey–Kramer adjusted *P* values are shown. LSD: *n* = 44; MSD: *n* = 52; HSD: *n* = 47. HSD, high-spice diet; LSD, low-spice diet; MSD, moderate-spice diet.

A main effect of diet was noted for the observed features (*P* = 0.046) α-diversity metric; no diet main effect was seen for Pielou's evenness ( *P* = 0.31) and Faith's phylogenetic diversity (*P* = 0.24; **Figure 1**). Post hoc pairwise testing showed that α-diversity based on the observed features metric was higher after the MSD than the LSD after adjustment for multiple comparisons (mean difference: 7.0 ASV; 95% CI: 0.2, 14.2; *P* = 0.057); no other pairwise tests approached significance. Compared with baseline, α-diversity assessed by Faith's phylogenetic diversity increased following the LSD (0.29 units; 95% CI: –0.01, 0.60; *P* = 0.057), MSD (0.50 units; 95% CI: 0.21, 0.79; *P* = 0.001), and HSD (0.32 units; 95% CI: 0.02, 0.62; *P* = 0.034). After the MSD, α-diversity assessed by the observed features metric was significantly greater than at baseline (11.2 ASV; 95% CI: 4.1, 18.3; *P* = 0.003); no differences were noted following the LSD (*P* = 0.35) or HSD (*P* = 0.19) as compared with baseline. Based on Pielou's evenness metric, no difference in α-diversity was seen between baseline and after the diets ( *P* > 0.1 for all).

**FIGURE 2:**
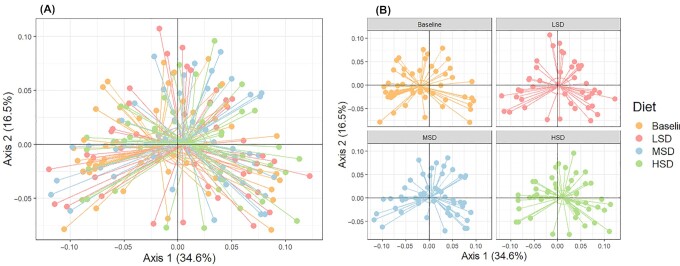
β-diversity assessed by principal coordinate analysis plots based on the weighted UniFrac distance metric (A) overall and (B) separated by diet in participants at risk of CVD. LSD: *n* = 44; MSD: *n* = 52; HSD: *n* = 47. CVD, cardiovascular disease; HSD, high-spice diet; LSD, low-spice diet; MSD, moderate-spice diet.

There were no between-diet differences in β-diversity ( *P* = 0.45). Compared with baseline, differences in β-diversity were observed following the LSD ( *P* = 0.02), MSD (*P* = 0.001), and HSD (*P* = 0.004; **Figure 2**).

### Taxonomic enrichment

LEfSe (LDA ≥ 2.0) between-diet comparisons showed enrichment of the Ruminococcaceae family after the HSD as compared with the MSD (LDA = 4.22, *P* = 0.03) and the LSD (LDA = 4.47, *P* = 0.004; **Table 2**). Following the LSD, the Prevotellaceae (LDA = 4.08, *P* = 0.038) and Peptostreptococcaceae (LDA = 3.55, *P* = 0.011) families were enriched when compared with the MSD, and the Coriobacteriaceae family (LDA = 3.19, *P* = 0.034) was enriched after the LSD when compared with the HSD. The Peptococcaceae family (LDA = 2.44, *P* = 0.044) was enriched following the MSD compared with the HSD. At the genus level, several members of the Ruminococcaceae family were enriched after the HSD as compared with the MSD and LSD. The *Agathobacter* genus was enriched following the HSD compared with the MSD (LDA = 4.03, *P* = 0.003) and LSD (LDA = 4.14, *P* = 0.045). After the LSD, the *Collinsella* genus was enriched compared with the HSD (LDA 3.19, *P* = 0.034).

**TABLE 2:** Between-diet comparisons of enriched bacteria in participants at risk of CVD^1^

Comparison^2^	Diet	LDA Score	Median RA, %	*P* Value
LSD compared with MSD				
D1_Bacteroidetes D2_Bacteroidia D3_Bacteroidales D4_Prevotellaceae	LSD	4.08	0	0.038
D1_Firmicutes D2_Clostridia D3_Clostridiales D4_Peptostreptococcaceae	LSD	3.55	1.20	0.011
D1_Firmicutes D2_Clostridia D3_Clostridiales D4_Peptostreptococcaceae D5_Romboutsia	LSD	3.47	0.84	0.047
D1_Firmicutes D2_Clostridia D3_Clostridiales D4_Peptostreptococcaceae D5_Romboutsia._	LSD	3.47	0.84	0.047
D1_Proteobacteria D2_Gammaproteobacteria	LSD	3.07	0.17	0.044
D1_Firmicutes D2_Clostridia D3_Clostridiales D4_Ruminococcaceae D5_Ruminococcus_1	MSD	3.28	1.12	0.045
D1_Firmicutes D2_Clostridia D3_Clostridiales D4_Lachnospiraceae D5_Lachnospiraceae_NK4A136_group D6_uncultured_organism	MSD	3.22	0.22	0.021
D1_Firmicutes D2_Clostridia D3_Clostridiales D4_Ruminococcaceae D5_Ruminiclostridium_6	MSD	3.01	0.03	0.045
D1_Bacteroidetes D2_Bacteroidia D3_Bacteroidales D4_Prevotellaceae D5_Paraprevotella	MSD	2.84	0.00	0.014
D1_Firmicutes D2_Clostridia D3_Clostridiales D4_Lachnospiraceae D5_Eubacterium_xylanophilum_group._	MSD	2.55	0.01	0.021
D1_Firmicutes D2_Clostridia D3_Clostridiales D4_Ruminococcaceae D5_Ruminiclostridium_6._	MSD	2.19	0.00	0.042
LSD compared with HSD				
D1_Firmicutes D2_Clostridia D3_Clostridiales D4_Lachnospiraceae D5_Eubacterium_hallii_group._	LSD	3.47	3.29	0.030
D1_Firmicutes D2_Clostridia D3_Clostridiales D4_Lachnospiraceae D5_Eubacterium_hallii_group	LSD	3.47	3.29	0.029
D1_Firmicutes D2_Clostridia D3_Clostridiales D4_Lachnospiraceae D5_Lachnoclostridium	LSD	3.23	0.27	0.019
D1_Actinobacteria D2_Coriobacteriia D3_Coriobacteriales D4_Coriobacteriaceae	LSD	3.19	0.43	0.034
D1_Actinobacteria D2_Coriobacteriia D3_Coriobacteriales D4_Coriobacteriaceae D5_0	LSD	3.19	0.43	0.034
D1_Actinobacteria D2_Coriobacteriia D3_Coriobacteriales D4_Coriobacteriaceae D5_Collinsella._	LSD	3.19	0.43	0.034
D1_Firmicutes D2_Clostridia D3_Clostridiales D4_Lachnospiraceae D5_Lachnoclostridium._	LSD	2.95	0.05	0.024
D1_Firmicutes D2_Clostridia D3_Clostridiales D4_Ruminococcaceae	HSD	4.47	24.90	0.004
D1_Firmicutes D2_Clostridia D3_Clostridiales	HSD	4.37	76.34	0.023
D1_Firmicutes D2_Clostridia	HSD	4.37	76.34	0.023
D1_Firmicutes D2_Clostridia D3_Clostridiales D4_Lachnospiraceae D5_Agathobacter	HSD	4.14	4.65	0.045
D1_Firmicutes D2_Clostridia D3_Clostridiales D4_Ruminococcaceae D5_Subdoligranulum	HSD	3.82	4.31	0.003
D1_Firmicutes D2_Clostridia D3_Clostridiales D4_Ruminococcaceae D5_Subdoligranulum._	HSD	3.80	4.31	0.003
D1_Firmicutes D2_Clostridia D3_Clostridiales D4_Ruminococcaceae D5_Ruminococcus_1	HSD	3.27	1.07	0.045
D1_Firmicutes D2_Clostridia D3_Clostridiales D4_Ruminococcaceae D5_Ruminiclostridium_6	HSD	2.90	0.03	0.040
D1_Firmicutes D2_Clostridia D3_Clostridiales D4_Ruminococcaceae D5_Ruminiclostridium_6 D6_uncultured_bacterium	HSD	2.88	0.00	0.029
D1_Firmicutes D2_Clostridia D3_Clostridiales D4_Lachnospiraceae D5_Eubacterium_xylanophilum_group._	HSD	2.56	0.05	0.005
D1_Firmicutes D2_Clostridia D3_Clostridiales D4_Lachnospiraceae D5_Eubacterium_xylanophilum_group	HSD	2.55	0.07	0.028
D1_Firmicutes D2_Clostridia D3_Clostridiales D4_Lachnospiraceae D5_Anaerosporobacter D6_uncultured_organism	HSD	2.42	0.00	0.042
MSD compared with HSD				
D1_Bacteroidetes D2_Bacteroidia D3_Bacteroidales D4_Bacteroidaceae D5_Bacteroides D6_Bacteroides_stercoris_ATCC_43183	MSD	3.06	0	0.044
D1_Tenericutes D2_Mollicutes	MSD	2.85	0	0.024
D1_Tenericutes	MSD	2.83	0	0.024
D1_Tenericutes D2_Mollicutes D3_Mollicutes_RF39	MSD	2.82	0	0.025
D1_Firmicutes D2_Clostridia D3_Clostridiales D4_Peptococcaceae	MSD	2.44	0	0.044
D1_Firmicutes D2_Clostridia D3_Clostridiales D4_Christensenellaceae D5_Christensenellaceae_R_7_group D6_uncultured_bacterium	MSD	2.12	0	0.045
D1_Firmicutes D2_Clostridia D3_Clostridiales D4_Ruminococcaceae	HSD	4.22	24.90	0.030
D1_Firmicutes D2_Clostridia D3_Clostridiales D4_Lachnospiraceae D5_Agathobacter._	HSD	4.04	4.55	0.002
D1_Firmicutes D2_Clostridia D3_Clostridiales D4_Lachnospiraceae D5_Agathobacter	HSD	4.03	4.65	0.003
D1_Firmicutes D2_Clostridia D3_Clostridiales D4_Ruminococcaceae D5_Subdoligranulum	HSD	3.68	4.31	0.037
D1_Firmicutes D2_Clostridia D3_Clostridiales D4_Ruminococcaceae D5_Subdoligranulum._	HSD	3.67	4.31	0.037
D1_Firmicutes D2_Clostridia D3_Clostridiales D4_Ruminococcaceae D5_Ruminococcaceae_UCG_002._	HSD	2.99	0.14	0.037
D1_Proteobacteria D2_Gammaproteobacteria D3_Enterobacteriales D4_Enterobacteriaceae._._	HSD	2.01	0	0.037
D1_Proteobacteria D2_Gammaproteobacteria D3_Enterobacteriales D4_Enterobacteriaceae._	HSD	2.01	0	0.037

1 LDA scores quantify the strength of enrichment within each categorical group. The *P* values were derived from Wilcoxon signed rank tests. B, baseline; CVD, cardiovascular disease; LDA, linear discriminant analysis; HSD, high-spice diet; LSD, low-spice diet; MSD, moderate-spice diet; RA, relative abundance.

2 D1: phylum; D2: class; D3: order; D4: family; D5: genus; D6: species.

**FIGURE 3:**
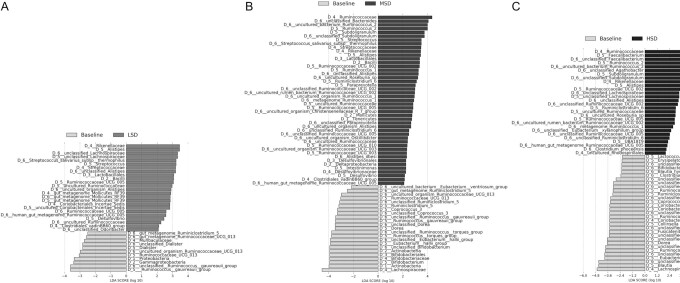
LEfSe enrichment plots display significantly (*P* ≤ 0.05, LDA ≥ 2) enriched taxa following the (A) LSD, (B) MSD, and (C) HSD when compared with baseline in participants at risk of CVD. LDA scores are displayed on the x-axis and quantify the strength of enrichment within each categorical group. LSD: *n* = 44; MSD: *n* = 52; HSD: *n* = 47. CVD, cardiovascular disease; HSD, high-spice diet; LDA, linear discriminant analysis; LEfSe, linear discriminant analysis effect size; LSD, low-spice diet; MSD, moderate-spice diet.

LEfSe comparisons of the spice-containing diets with baseline showed that multiple taxa were differentially abundant following the spice-containing diets (**Figure 3**, **Supplemental Table 6**). Compared with baseline, the Ruminococcaceae family was enriched after the HSD (LDA = 4.62, *P* < 0.001) and MSD (LDA = 4.36, *P* = 0.004). The Rikenellaceae family was enriched after the HSD (LDA = 3.44, *P* = 0.008), MSD (LDA = 3.44, *P* < 0.001), and LSD (LDA = 3.46, *P* = 0.017) when compared with baseline. At the genus level, several members of the Ruminococcaceae family were enriched after the HSD as compared with baseline: *Faecalibacterium* (LDA = 4.25, *P* = 0.002), *Ruminococcus 2* (LDA = 4.11, *P* < 0.001), and *Ruminococcaceae UCG005* (LDA = 2.89, *P* = 0.006). Enrichment of the *Odoribacter* species (LDA = 2.08, *P* = 0.018) was observed after the LSD as compared with baseline.

### Predictive functional biomarker analysis

PICRUSt2 analyses showed several functional pathways predicted to be enriched after the HSD as compared with the MSD and LSD (**Table 3**). Many of the pathways predicted to be enriched after the HSD were related to genetic information processing, cellular processes, and nucleotide and amino acid metabolism. The *K03205* gene was predicted to be enriched after the HSD as compared with the MSD (LDA = 2.08, *P* = 0.011) and LSD (LDA = 2.17, *P* = 0.011). No genes were predicted to be enriched after the MSD as compared with LSD. Differential predicted enrichment of several functional pathways and genes was noted following the spice-containing diets as compared with baseline (**Supplemental Tables 7** and **8**).

**TABLE 3:** Between-diet differences in predictive functional pathways in participants at risk of CVD^1^

Comparison	Diet	LDA Score	*P* Value
LSD compared with MSD			
None	—	—	—
LSD compared with HSD			
Metabolism	LSD	3.28	0.011
Metabolism of cofactors and vitamins	LSD	2.61	0.012
Fructose and mannose metabolism PATH ko00051	LSD	2.47	0.047
Folate biosynthesis PATH ko00790	LSD	2.32	0.001
Carbon fixation pathways in prokaryotes PATH ko00720	LSD	2.10	0.003
Cellular processes	HSD	2.83	0.022
Cell motility	HSD	2.80	0.021
Flagellar assembly PATH ko02040	HSD	2.61	0.041
Signal transduction	HSD	2.48	0.033
Two-component system PATH ko02020	HSD	2.45	0.044
Bacterial secretion system PATH ko03070	HSD	2.40	0.037
Bacterial chemotaxis PATHko02030	HSD	2.36	0.047
Peptidoglycan biosynthesis PATH ko00550	HSD	2.19	0.045
Photosynthesis PATH ko00195	HSD	2.14	0.050
Cysteine and methionine metabolism PATH ko00270	HSD	2.06	0.031
MSD compared with HSD			
Metabolism	MSD	3.06	0.024
Fructose and mannose metabolism PATH ko00051	MSD	2.51	0.024
Folate biosynthesis PATH ko00790	MSD	2.15	0.003
Propanoate metabolism PATH ko00640	MSD	2.06	0.025
Arginine biosynthesis PATH ko00220	MSD	2.06	0.011
Cellular processes	HSD	2.85	0.026
Flagellar assembly PATH ko02040	HSD	2.62	0.034
Bacterial secretion system PATH ko03070	HSD	2.29	0.031

1 LEfSe analyses display predicted enrichment (*P* ≤ 0.05, LDA ≥2) of functional pathways based on PICRUSt2-predicted KEGG orthologs between the diets. The *P* values were derived from Wilcoxon signed rank tests. CVD, cardiovascular disease; LDA, linear discriminant analysis; LEfSe, linear discriminant analysis effect size; HSD, high-spice diet; KEGG, Kyoto Encyclopedia of Genes and Genomes; LSD, low-spice diet; MSD, moderate-spice diet; PICRUSt2, Phylogenetic Investigation of Communities by Reconstruction of Unobserved States 2.

## Discussion

In this study, we evaluated shifts in gut bacterial composition following intake of an average American diet with 3 doses of herbs and spices in adults at risk for CVD. Our findings suggest bacterial metabolism of culinary doses of herbs and spices when consumed as part of a well-defined constant background diet. We saw differences in α-diversity between the LSD and MSD, as well as the HSD and MSD, compared with baseline. No difference in β-diversity was seen between the diets. Differences in taxonomic enrichment occurred between the diets. The Ruminococcaceae family was enriched after the HSD as compared with the MSD and LSD, as well as after the HSD and MSD as compared with baseline. These findings suggest that culinary doses of herbs and spices modulate gut bacterial composition within 4 wk in adults at increased risk of CVD.

α-diversity, or ASV richness, was higher according to the observed features metric following the MSD when compared with the LSD. However, based on the Pielou's evenness α-diversity metric, no between-diet differences were noted. This suggests that the MSD increased the number of ASVs present but not the uniformity of those ASVs’ abundances, possibly because 24 herbs and spices were incorporated into the 7-d menu in relatively small doses; therefore, a limited amount of substrate was available for bacterial metabolism. In further support of herb/spice-induced increases in species richness, a tendency toward a dose–response increase in α-diversity after the spice diets as compared with baseline was apparent according to Faith's phylogenetic diversity metric. However, these within-diet findings should be interpreted with caution because prior to baseline testing, participants were consuming their habitual diets, not the standardized study diet. Substantial variation was seen with all 3 α-diversity metrics, which likely attenuated the results for the between-diet comparisons toward the null.

Our α-diversity results contrast with Lu and colleagues’ (5) findings of no effect of a 5-g capsule containing herbs and spices (cinnamon: 20%; oregano: 30%; ginger: 30%; black pepper: 17%; cayenne pepper: 3%) on α-diversity after 2 wk when compared with a maltodextrin placebo. The HSD in the present study contained a similar amount of cinnamon to the capsule tested by Lu et al., although the doses of oregano, ginger, and black pepper were much lower in the present study and cayenne pepper was not used. Thus, the divergent findings may be explained by differences in the study duration (4 wk compared with 2 wk), the study population (adults at risk of CVD compared with healthy adults), the spice delivery (part of a diet compared with a capsule), the herb/spice composition (24 compared with 5 unique herbs/spices), or doses tested (0.5, 3.3, and 6.6 g.d^–1^.2100 kcal^–1^ compared with 5 g/d).

We saw differences in β-diversity following the spice-containing diets as compared with baseline, although these findings should be interpreted cautiously because they may reflect a change in the background diets rather than the presence of herbs and spices in the study diets. The lack of between-diet differences in β-diversity in our study aligns with Lu and colleagues’ (5) β-diversity findings.

At a taxonomic level, the Ruminococcaceae family was enriched after the HSD as compared with the MSD and LSD and after the MSD and HSD as compared with baseline. In alignment, Lu et al. (5) saw enrichment of 4 OTUs within the Ruminococcaceae family. An analysis of healthy females from the TwinsUK registry showed OTUs assigned to the Ruminococcaceae family were associated with lower long-term weight gain (median follow-up: 9 y) after adjustment for age, sex, smoking, calorie intake, physical activity, and family relatedness (23). In mice, changes in gut bacterial composition including enrichment of *Clostridia* from the Mogibacteriaceae and Ruminococcaceae families were shown to contribute to suppression of diet-induced obesity with exposure to cold temperature (12 °C) (24). This suggests that gut bacteria contribute to metabolic pathways that increase energy expenditure to protect against diet-induced obesity in response to cold exposure.

Capsinoids, found in red peppers, have been shown to increase activation of brown adipose tissue and increase energy expenditure, although to a lesser extent than cold exposure (25). Preclinical studies suggest a role of gut bacteria in capsinoid-induced increases in energy expenditure and protection against diet-induced obesity (26). Therefore, the enrichment of the Ruminococcaceae family observed with the HSD may be in part a response to the capsinoid content of the diet and may confer a lean phenotype. In the present study, the diets were designed for weight maintenance, and there were no between-diet differences in body weight, although after all 3 diets, body weight was slightly reduced from baseline (0.8–1.1 kg). Therefore, future studies are needed to examine the role of herb/spice intake in bacterial-related changes in energy expenditure, body weight regulation, and obesity.

We saw enrichment of the *Agathobacter* genus after the HSD as compared with the MSD and LSD, and Faecalibacterium was enriched after the HSD when compared with baseline. *Faecalibacterium* and *Agathobacter* are known to produce SCFAs, such as butyrate and propionic acid. Butyrate is the primary energy source for colonocytes (27) and is essential for intestinal epithelium maintenance, barrier function, and regulation of cell turnover (28). SCFAs also exert anti-inflammatory effects in the intestinal mucosa (29). In alignment, we saw reductions in proinflammatory cytokines with the spice-containing diets from baseline (8). Therefore, it is possible that changes in the gut bacteria mediated the spice-induced anti-inflammatory effects.

The analyses conducted to predict the functional capabilities of the enriched gut bacteria revealed predicted enrichment of several functional pathways and 1 functional gene after the HSD as compared with the MSD and LSD, which are related to genetic information processing, cellular processes, and nucleotide and amino acid metabolism. Following the HSD compared with baseline, several functional pathways related to genetic information processing were predicted to be enriched, including the mismatch repair pathway, which has a key role in maintaining genetic stability. Herbs and spices are known to have antioxidant properties, such as high free radical scavenging activity and reducing power (30). In addition, herbs and spices exert anti-inflammatory effects (31), and we observed enrichment of *Faecalibacterium* and *Agathobacter*, SCFA-producing bacteria, which may contribute to reducing intestinal inflammation. Further investigation is needed to confirm these predictive analyses.

In this randomized controlled feeding study, the effect of 4 wk of exposure to mixed herbs and spices on gut bacteria composition was evaluated, to our knowledge, for the first time. Strengths of the study include the controlled feeding design whereby known doses of herbs and spices were provided in the context of a constant, well-characterized background diet, therefore minimizing confounding from other dietary factors, particularly dietary fiber. In addition, self-reported adherence was high. However, this study is limited by the exploratory nature of the analyses, inclusion of a subset of the original study sample that consented to participate in this substudy, and an inflated risk of type I statistical errors from testing multiple outcomes. In addition, we did not screen participants based on prior antibiotic or probiotic use, which may affect gut microbiota composition. Only 1 participant reported taking antibiotics during the study (in diet period 1); information on probiotic supplement use was not collected. Furthermore, 24 herbs and spices were included in the test menus, which limits inferences about the effect of individual ones. In addition, each day in the 7-d menu included different combinations and amounts of the 24 herbs and spices; therefore, a consistent daily exposure was not provided. This study is also limited by the lack of data about habitual herb/spice intake at baseline and the lack of chemical analysis to verify the composition of the test herbs and spices. Prior research has documented differences in microbiota composition in individuals with obesity as well as other risk factors for CVD (6); thus, the findings from this sample of adults at risk for CVD need to be confirmed in a sample of healthy adults. Finally, PICRUSt2 was used to predict functional implications of the changes in gut bacterial composition; as such, these results need to be confirmed by metatranscriptomics.

In conclusion, the findings of this prespecified exploratory analysis showed compositional shifts in gut bacteria following intake of an average American diet with 3 doses of herbs and spices for 4 wk in adults at risk for CVD. In response to the increasing doses of herbs and spices, there was a tendency toward greater ASV diversity. Furthermore, after a US-style dietary pattern with a high culinary dose of mixed herbs and spices (6.6 g.d^–1^.2100 kcal^–1^), the Ruminococcaceae family was enriched when compared with matched dietary patterns with lower doses (0.5 and 3.3 g.d^–1^.2100 kcal^–1^). This study suggests that incorporation of culinary doses of herbs and spices into an average American diet changes gut bacterial composition in adults at risk for CVD. Further investigation of the metabolic implications of these bacterial changes is needed.

## Supplementary Material

nxac201_Supplemental_File

## Data Availability

Data are available at https://www.ncbi.nlm.nih.gov/sra, BioProject PRJNA844583.

## Notes

This study was funded by the McCormick Science Institute. In addition, the study was supported by the National Center for Advancing Translational Sciences, NIH, through grant 1UL1TR002014-01. The McCormick Science Institute and the NIH had no role in study design, data collection, data analysis, data interpretation, or writing of the manuscript. Chefs from McCormick and Company, Inc, designed the menus based on nutrient targets defined by the investigators. The content is solely the responsibility of the authors and does not necessarily represent the official views of the NIH. The study also received support for computational resources from the Howard Hughes Medical Institute through the Precollege and Undergraduate Science Education Program, as well as the National Science Foundation through awards DBI-1248096 and CBET-1805549.

Author disclosures: KSP, PMK-E, and RL received funding from the McCormick Science Institute for the research reported in this article. SA, JL, and JRCS report no conflicts of interest. Financial supporters had no role in data collection, analysis, and interpretation or in preparation, review, or approval of the manuscript.

Supplemental Tables 1–8 and Supplemental Figure 1 are available at the “Supplementary data” link in the online posting of the article and from the same link in the online table of contents at https://academic.oup.com/jn/.

Abbreviations used: ASV, amplicon sequence variant; hs-CRP, high-sensitivity CRP; HSD, high-spice diet; KEGG, Kyoto Encyclopedia of Genes and Genomes; LDA, linear discriminant analysis; LEfSe, linear discriminant analysis effect size; LSD, low-spice diet; MSD, moderate-spice diet; OTU, operational taxonomic unit; PICRUSt2, Phylogenetic Investigation of Communities by Reconstruction of Unobserved States 2.
