# Supplementary material for: Herbs and Spices Modulate Gut Bacterial Composition in Adults at Risk for CVD: Results of a Prespecified Exploratory Analysis from a Randomized, Crossover, Controlled-Feeding Study
Source: J Nutr. 2022 Sep 2;152(11):2461–70. doi: 10.1093/jn/nxac201 (PMC9644184; doi:10.1093/jn/nxac201)
Supplement: nxac201_Supplemental_File [file nxac201_supplemental_file.docx]

# Online Supplementary Material

## Supplemental Table 1: The nutrient composition of the background study diet

| Nutrient^1^ |  |
| --- | --- |
| Carbohydrate, % energy | 50 |
| Protein, % energy | 17 |
| Total fat, % energy | 33 |
| Saturated fat, % energy | 11 |
| Monounsaturated fat, % energy | 11 |
| Polyunsaturated fat, % energy | 8 |
| Fiber, g/d | 22 |
| Sodium, mg/d | 3023 |

Nutrient composition was estimated using Food Processor® (ESHA Research)

^1^Based on the 2100 kcal diet

## Supplemental Table 2: The herb and spice composition of the study diets at 2100 kcal

| **Herb/Spice g/d ± SD (% of total dose)^1^** | **LSD** | **MSD** | **HSD** |
| --- | --- | --- | --- |
|  | **g/d ± SD (% of total dose)^1^** | | |
| Cinnamon | 0.099 ± 0.033 (18.55) | 0.595 ± 0.199 (18.51) | 1.190 ± 0.398 (18.51) |
| Coriander | 0.069 ± 0.027 (12.98) | 0.417 ± 0.163 (12.96) | 0.833 ± 0.325 (12.96) |
| Ginger | 0.055 ± 0.065 (10.23) | 0.328 ± 0.391(10.21) | 0.656 ± 0.781 (10.21) |
| Cumin | 0.045 ± 0.029 (8.42) | 0.270 ± 0.175 (8.40) | 0.540 ± 0.351 (8.40) |
| Parsley | 0.041 ± 0.034 (7.72) | 0.238 ± 0.184 (7.42) | 0.477 ± 0.368 (7.42) |
| Black pepper | 0.039 ± 0.021 (7.36) | 0.239 ± 0.135 (7.45) | 0.479 ± 0.027 (7.45) |
| Garlic | 0.028 ± 0.031 (5.25) | 0.174 ± 0.198 (5.42) | 0.348 ± 0.396 (5.42) |
| Turmeric | 0.026 ± 0.031 (4.88) | 0.156 ± 0.184 (4.87) | 0.313 ± 0.369 (4.87) |
| Onion powder | 0.026 ± 0.017 (4.85) | 0.156 ± 0.104 (4.85) | 0.311 ± 0.208 (4.85) |
| Paprika | 0.020 ± 0.030 (3.80) | 0.122 ± 0.178 (3.79) | 0.244 ± 0.356 (3.79) |
| Chili powder | 0.014 ± 0.026 (2.67) | 0.086 ± 0.157 (2.67) | 0.171 ± 0.315 (2.67) |
| Rosemary | 0.013 ± 0.009 (2.41) | 0.080 ± 0.057 (2.50) | 0.161 ± 0.114 (2.50) |
| Cilantro | 0.013 ± 0.011 (2.38) | 0.076 ± 0.069 (2.38) | 0.153 ± 0.137 (2.38) |
| Oregano | 0.013 ± 0.013 (2.35) | 0.075 ± 0.080 (2.35) | 0.151 ± 0.160 (2.35) |
| Basil | 0.011 ± 0.012 (2.13) | 0.068 ± 0.075 (2.13) | 0.137 ± 0.150 (2.13) |
| Red pepper | 0.009 ± 0.007 (1.59) | 0.051 ± 0.042 (1.59) | 0.102 ± 0.084 (1.59) |
| Thyme | 0.008 ± 0.007 (1.50) | 0.050 ± 0.045 (1.57) | 0.101 ± 0.090 (1.57) |
| Bayleaf | 0.006 ± 0.008 (1.21) | 0.040 ± 0.047 (1.25) | 0.080 ± 0.094 (1.25) |
| Cardamom | 0.004 ± 0.006 (0.76) | 0.024 ± 0.034 (0.76) | 0.049 ± 0.069 (0.76) |
| Sesame seeds | 0.002 ± 0.006 (0.44) | 0.014 ± 0.038 (0.44) | 0.029 ± 0.076 (0.44) |
| Sage | 0.002 ± 0.005 (0.33) | 0.011 ± 0.028 (0.33) | 0.021 ± 0.057 (0.33) |
| Poppy seeds | 0.001 ± 0.003 (0.22) | 0.007 ± 0.019 (0.22) | 0.014 ± 0.038 (0.22) |
| Dill weed | <0.001 ± 0.001 (0.08) | 0.003 ± 0.007 (0.08) | 0.005 ± 0.014 (0.08) |
| Allspice | <0.001 ± 0.001 (0.08) | 0.003 ± 0.007 (0.08) | 0.005 ± 0.013 (0.08) |
| Total | 0.547 ± 0.067 | 3.285 ± 0.417 | 6.571 |

^1^Values represent the mean composition of the 7 days included in the menu ± SD; The herbs and spices used were in the dried form. LSD, low spice diet; MSD, moderate spice diet; HSD, high spice diet

## Supplemental Table 3: The seven-day menu used in the study including the added herbs/spices^1^

| **Meal** | **Menu Day** | | | | | | |
| --- | --- | --- | --- | --- | --- | --- | --- |
|  | **1** | **2** | **3** | **4** | **5** | **6** | **7** |
| **BREAKFAST** | **Bagel with spiced cream cheese** *(black pepper, garlic, oregano, poppy seeds, sesame seeds)*  **Banana**  **Apple juice** | **Oatmeal with applesauce** *(cinnamon, ginger)* **Apple and cran-apple juice** | **Life cereal**  *(cinnamon)*  **Banana**  **Orange juice** | **Parfait, fruit and yogurt** *(cinnamon, ginger)*  **Apple juice** | **Fruit smoothie** *(ginger)*  **Banana** | **Veggie, cheese egg scramble** *(black pepper)*  **English muffin**  **Orange juice** | **Buttermilk pancakes with syrup**  **Orange juice** |
| **LUNCH** | **Chicken salad sandwich** *(bayleaf, cardamom, cilantro, cinnamon, coriander, cumin, garlic, ginger, onion, red pepper, turmeric)*  **Apple** | **Grilled chicken** *(black pepper, garlic, onion, paprika, turmeric)*  **Rice** *(cardamom, cinnamon, coriander, ginger)*  **Dressing** *(ginger)* | **White chicken chili** *(black pepper, cilantro, coriander, cumin, red pepper)*  **Cornbread** | **Grilled chicken sandwich with**  **dijonnaise** *(black pepper, coriander, onion, parsley, rosemary, turmeric)*  **Grapes** | **Chicken quesadilla** *(cilantro, coriander, cumin, chili, red pepper)* | **Couscous salad** *(cilantro, cinnamon, coriander,*  *cumin, garlic, ginger, onion,*  *red pepper,*  *turmeric)*  **Grilled chicken** | **Chef’s salad** *(black pepper, turmeric, coriander)* |
| **DINNER** | **Steak tips** *(black pepper, onion, rosemary, thyme)*  **Gravy** *(black pepper, onion, rosemary, thyme)*  **Mashed potato** *(garlic, onion, parsley)*  **Steamed broccoli** *(black pepper, coriander, dill weed, garlic, onion, red pepper, turmeric)* | **Turkey stroganoff** *(basil, bayleaf, black pepper, cinnamon, rosemary, sage, thyme)*  **Egg noodles** *(parsley)* | **Chicken Etouffee** *(bayleaf, black pepper, garlic, onion, oregano, red pepper, thyme)*  **Rice** *(parsley)*  **Side salad + dressing** *(basil, black pepper, oregano, parsley)* | **Beef tacos** *(red pepper, coriander, cumin, chili, paprika)*  **Corn and peas** *(cilantro, coriander, cumin, onion, paprika)* | **Marinara sauce** *(basil, bayleaf, garlic, onion, oregano, parsley)*  **Meatballs** *(basil, black pepper, garlic, parsley, rosemary, thyme)*  **Spaghetti**  **Side salad + dressing** *(basil, black pepper, oregano, parsley)* | **Meatloaf** *(bayleaf, black pepper, garlic, parsley, rosemary, thyme)*  **Mashed potatoes** *(garlic, onion, parsley)*  **Steamed green beans** *(black pepper, coriander, garlic, onion, parsley)* | **Tikka Masala** **Sauce** *(cardamom, coriander, cumin, ginger, paprika, red pepper, turmeric)*  **Chicken** *(cumin, garlic, paprika, turmeric)*  **Basmati Rice**  **Naan** |
| **SNACKS** | **Crudité with sour cream dip** *(basil, black pepper, coriander, parsley)* | **Hummus** *(coriander, cumin, turmeric)*  **Whole wheat pita** | **Dinner roll with spiced butter** *(black pepper, coriander, parsley, rosemary)* | **Cracker sandwich with cheese filling** | **Queso dip** *(coriander, cumin, oregano, red pepper, turmeric)*  **Tortilla chips** | **Sundried tomato spread** *(basil, black pepper, coriander, garlic, onion, oregano)*  **Crostini** | **Chex mix** *(coriander, cumin, turmeric)* |
|  | **Apple pie yoghurt parfait** *(cinnamon)* | **Raisinets** | **Apple**  **Spiced peanut butter** *(cinnamon)* | **Soft pretzel Cream cheese dip** *(allspice, cinnamon, ginger)* | **Oatmeal raisin cookies** *(cinnamon)* | **Apple pie yogurt parfait** *(cinnamon)* | **Banana bread** *(cinnamon)* |

^1^The same menu was used for all of the test diets; the only difference between the diets was the quantity of spices included in spice- containing recipes. Herbs/spices listed in parentheses were added to the recipe.

## Supplemental Table 4: Baseline characteristics of the entire cohort and the sub-study cohort

| **Characteristics** | **Entire cohort**  **(n=71)** | **Sub-Study Cohort**  **(n=54)** |
| --- | --- | --- |
| Sex, n (%) |  |  |
| Male | 32 (45) | 23 (43) |
| Female | 39 (55) | 31 (57) |
| Age, years | 44 ± 11 | 45 ± 11 |
| BMI, kg/m^2^ | 29.7 ± 2.9 | 29.8 ± 2.9 |
| Waist circumference, cm | 102.4 ± 7.3 | 102.8 ± 7.1 |
| Male | 103.4 ± 6.9 | 103.2 ± 6.0 |
| Female | 101.6 ± 7.7 | 102.5 ± 7.8 |
| Total Cholesterol, mg/dL | 193 ± 34 | 193 ± 33 |
| LDL-cholesterol, mg/dL | 126 ± 28 | 126 ± 28 |
| HDL-cholesterol, mg/dL | 48 ± 12 | 49 ± 11 |
| Triglycerides, mg/dL | 106 (79, 149)^1^ | 105 (79-117)^1^ |
| Glucose^2^, mg/dL | 100 ± 7 | 99 ± 7 |
| hs-CRP, mg/L | 2.3 (0.8, 4.0)^1,3^ | 2.5 (1.0-4.6)^1,4^ |
| Systolic blood pressure, mmHg | 129 ± 14 | 129 ± 13 |
| Diastolic blood pressure, mmHg | 81 ± 11 | 81 ± 10 |

Data presented as mean ± standard deviation unless otherwise stated; Biological analytes were measured in serum unless otherwise stated; ^1^ Data presented as median (interquartile range); ^2^ Measured in plasma; ^3^ Values >10 mg/L were excluded from analysis, n=68; ^4^ Values >10 mg/L were excluded from analysis, n=52

## Supplemental Table 5: Forward and Reverse Sequence Quality Data

| Position (BP) | Forward Reads Median Expected Error | Forward Reads Mean Expected Error | Forward Reads 75% Percentile Expected Error | Reverse Reads Median Expected Error | Reverse Reads Mean Expected Error | Reverse Reads 75% Percentile Expected Error |
| --- | --- | --- | --- | --- | --- | --- |
| 1 | 0 | 0 | 0 | 0 | 0 | 0 |
| 2 | 0 | 0 | 0 | 0 | 0 | 0 |
| 3 | 0 | 0 | 0 | 0 | 0 | 0 |
| 4 | 0 | 0 | 0 | 0 | 0.01 | 0 |
| 5 | 0 | 0.01 | 0 | 0 | 0.01 | 0 |
| 6 | 0 | 0.01 | 0 | 0 | 0.01 | 0 |
| 7 | 0 | 0.01 | 0 | 0 | 0.01 | 0 |
| 8 | 0 | 0.01 | 0 | 0 | 0.01 | 0 |
| 9 | 0 | 0.01 | 0.01 | 0 | 0.01 | 0 |
| 10 | 0 | 0.01 | 0.01 | 0 | 0.01 | 0.01 |
| 11 | 0 | 0.01 | 0.01 | 0 | 0.01 | 0.01 |
| 12 | 0.01 | 0.01 | 0.01 | 0 | 0.02 | 0.01 |
| 13 | 0.01 | 0.01 | 0.02 | 0 | 0.02 | 0.01 |
| 14 | 0.01 | 0.02 | 0.02 | 0 | 0.02 | 0.01 |
| 15 | 0.01 | 0.02 | 0.02 | 0 | 0.02 | 0.01 |
| 16 | 0.01 | 0.02 | 0.02 | 0.01 | 0.02 | 0.01 |
| 17 | 0.01 | 0.02 | 0.02 | 0.01 | 0.02 | 0.01 |
| 18 | 0.01 | 0.02 | 0.02 | 0.01 | 0.02 | 0.01 |
| 19 | 0.01 | 0.02 | 0.02 | 0.01 | 0.02 | 0.01 |
| 20 | 0.01 | 0.02 | 0.02 | 0.01 | 0.02 | 0.02 |
| 21 | 0.01 | 0.02 | 0.02 | 0.01 | 0.03 | 0.02 |
| 22 | 0.01 | 0.02 | 0.02 | 0.01 | 0.03 | 0.02 |
| 23 | 0.01 | 0.02 | 0.02 | 0.01 | 0.03 | 0.02 |
| 24 | 0.01 | 0.02 | 0.02 | 0.01 | 0.03 | 0.02 |
| 25 | 0.01 | 0.02 | 0.02 | 0.01 | 0.03 | 0.02 |
| 26 | 0.01 | 0.02 | 0.03 | 0.01 | 0.03 | 0.02 |
| 27 | 0.01 | 0.03 | 0.03 | 0.01 | 0.03 | 0.02 |
| 28 | 0.01 | 0.03 | 0.03 | 0.01 | 0.03 | 0.02 |
| 29 | 0.01 | 0.03 | 0.03 | 0.01 | 0.03 | 0.02 |
| 30 | 0.01 | 0.03 | 0.03 | 0.01 | 0.04 | 0.02 |
| 31 | 0.01 | 0.03 | 0.03 | 0.01 | 0.04 | 0.03 |
| 32 | 0.01 | 0.03 | 0.03 | 0.01 | 0.04 | 0.03 |
| 33 | 0.01 | 0.03 | 0.03 | 0.01 | 0.04 | 0.03 |
| 34 | 0.01 | 0.03 | 0.03 | 0.01 | 0.04 | 0.03 |
| 35 | 0.01 | 0.03 | 0.03 | 0.01 | 0.04 | 0.03 |
| 36 | 0.01 | 0.03 | 0.03 | 0.01 | 0.04 | 0.03 |
| 37 | 0.01 | 0.03 | 0.03 | 0.01 | 0.04 | 0.03 |
| 38 | 0.01 | 0.03 | 0.04 | 0.01 | 0.04 | 0.03 |
| 39 | 0.01 | 0.04 | 0.04 | 0.01 | 0.05 | 0.03 |
| 40 | 0.01 | 0.04 | 0.04 | 0.01 | 0.05 | 0.03 |
| 41 | 0.01 | 0.04 | 0.04 | 0.01 | 0.05 | 0.04 |
| 42 | 0.01 | 0.04 | 0.04 | 0.01 | 0.05 | 0.04 |
| 43 | 0.01 | 0.04 | 0.04 | 0.01 | 0.05 | 0.04 |
| 44 | 0.01 | 0.04 | 0.04 | 0.01 | 0.05 | 0.04 |
| 45 | 0.01 | 0.04 | 0.04 | 0.01 | 0.06 | 0.04 |
| 46 | 0.01 | 0.04 | 0.04 | 0.01 | 0.06 | 0.04 |
| 47 | 0.01 | 0.04 | 0.04 | 0.01 | 0.06 | 0.04 |
| 48 | 0.01 | 0.04 | 0.05 | 0.01 | 0.06 | 0.04 |
| 49 | 0.01 | 0.05 | 0.05 | 0.01 | 0.06 | 0.05 |
| 50 | 0.01 | 0.05 | 0.05 | 0.01 | 0.06 | 0.05 |
| 51 | 0.01 | 0.05 | 0.05 | 0.01 | 0.06 | 0.05 |
| 52 | 0.02 | 0.05 | 0.05 | 0.01 | 0.07 | 0.05 |
| 53 | 0.02 | 0.05 | 0.05 | 0.01 | 0.07 | 0.05 |
| 54 | 0.02 | 0.05 | 0.05 | 0.01 | 0.07 | 0.05 |
| 55 | 0.02 | 0.05 | 0.05 | 0.01 | 0.07 | 0.05 |
| 56 | 0.02 | 0.05 | 0.06 | 0.01 | 0.07 | 0.05 |
| 57 | 0.02 | 0.06 | 0.06 | 0.01 | 0.07 | 0.06 |
| 58 | 0.02 | 0.06 | 0.07 | 0.01 | 0.07 | 0.06 |
| 59 | 0.02 | 0.06 | 0.07 | 0.01 | 0.08 | 0.06 |
| 60 | 0.02 | 0.06 | 0.07 | 0.01 | 0.08 | 0.06 |
| 61 | 0.02 | 0.07 | 0.07 | 0.01 | 0.08 | 0.06 |
| 62 | 0.02 | 0.07 | 0.07 | 0.01 | 0.08 | 0.06 |
| 63 | 0.02 | 0.07 | 0.07 | 0.02 | 0.08 | 0.06 |
| 64 | 0.03 | 0.07 | 0.07 | 0.02 | 0.08 | 0.06 |
| 65 | 0.03 | 0.07 | 0.08 | 0.02 | 0.08 | 0.06 |
| 66 | 0.03 | 0.07 | 0.08 | 0.02 | 0.09 | 0.07 |
| 67 | 0.03 | 0.07 | 0.08 | 0.02 | 0.09 | 0.07 |
| 68 | 0.03 | 0.07 | 0.08 | 0.02 | 0.09 | 0.07 |
| 69 | 0.03 | 0.07 | 0.08 | 0.02 | 0.09 | 0.07 |
| 70 | 0.03 | 0.08 | 0.08 | 0.02 | 0.09 | 0.07 |
| 71 | 0.03 | 0.08 | 0.08 | 0.02 | 0.09 | 0.07 |
| 72 | 0.03 | 0.08 | 0.08 | 0.02 | 0.09 | 0.07 |
| 73 | 0.03 | 0.08 | 0.08 | 0.02 | 0.09 | 0.07 |
| 74 | 0.03 | 0.08 | 0.08 | 0.02 | 0.1 | 0.07 |
| 75 | 0.03 | 0.08 | 0.08 | 0.02 | 0.1 | 0.07 |
| 76 | 0.03 | 0.08 | 0.09 | 0.02 | 0.1 | 0.07 |
| 77 | 0.03 | 0.08 | 0.09 | 0.02 | 0.1 | 0.08 |
| 78 | 0.03 | 0.08 | 0.09 | 0.03 | 0.1 | 0.08 |
| 79 | 0.03 | 0.09 | 0.09 | 0.03 | 0.1 | 0.08 |
| 80 | 0.03 | 0.09 | 0.09 | 0.03 | 0.11 | 0.08 |
| 81 | 0.03 | 0.09 | 0.1 | 0.03 | 0.11 | 0.08 |
| 82 | 0.03 | 0.09 | 0.1 | 0.03 | 0.11 | 0.08 |
| 83 | 0.03 | 0.09 | 0.1 | 0.03 | 0.11 | 0.08 |
| 84 | 0.03 | 0.09 | 0.1 | 0.03 | 0.11 | 0.08 |
| 85 | 0.03 | 0.09 | 0.1 | 0.03 | 0.11 | 0.09 |
| 86 | 0.03 | 0.1 | 0.1 | 0.03 | 0.11 | 0.09 |
| 87 | 0.03 | 0.1 | 0.1 | 0.03 | 0.11 | 0.09 |
| 88 | 0.03 | 0.1 | 0.1 | 0.03 | 0.11 | 0.09 |
| 89 | 0.03 | 0.1 | 0.1 | 0.03 | 0.12 | 0.09 |
| 90 | 0.03 | 0.1 | 0.1 | 0.03 | 0.12 | 0.09 |
| 91 | 0.03 | 0.1 | 0.11 | 0.03 | 0.12 | 0.09 |
| 92 | 0.04 | 0.1 | 0.11 | 0.03 | 0.12 | 0.09 |
| 93 | 0.04 | 0.11 | 0.11 | 0.03 | 0.12 | 0.09 |
| 94 | 0.04 | 0.11 | 0.11 | 0.03 | 0.12 | 0.09 |
| 95 | 0.04 | 0.11 | 0.12 | 0.03 | 0.12 | 0.09 |
| 96 | 0.04 | 0.11 | 0.12 | 0.03 | 0.12 | 0.09 |
| 97 | 0.04 | 0.11 | 0.12 | 0.03 | 0.12 | 0.1 |
| 98 | 0.04 | 0.12 | 0.12 | 0.03 | 0.13 | 0.1 |
| 99 | 0.04 | 0.12 | 0.12 | 0.03 | 0.13 | 0.1 |
| 100 | 0.04 | 0.12 | 0.12 | 0.03 | 0.13 | 0.1 |
| 101 | 0.04 | 0.12 | 0.12 | 0.03 | 0.13 | 0.1 |
| 102 | 0.04 | 0.12 | 0.13 | 0.04 | 0.13 | 0.1 |
| 103 | 0.04 | 0.12 | 0.13 | 0.04 | 0.13 | 0.1 |
| 104 | 0.04 | 0.13 | 0.13 | 0.04 | 0.14 | 0.11 |
| 105 | 0.04 | 0.13 | 0.13 | 0.04 | 0.14 | 0.11 |
| 106 | 0.04 | 0.13 | 0.13 | 0.04 | 0.14 | 0.11 |
| 107 | 0.04 | 0.13 | 0.13 | 0.04 | 0.14 | 0.11 |
| 108 | 0.04 | 0.13 | 0.13 | 0.04 | 0.14 | 0.11 |
| 109 | 0.04 | 0.13 | 0.14 | 0.04 | 0.15 | 0.11 |
| 110 | 0.04 | 0.13 | 0.14 | 0.04 | 0.15 | 0.12 |
| 111 | 0.04 | 0.14 | 0.14 | 0.04 | 0.15 | 0.12 |
| 112 | 0.04 | 0.14 | 0.14 | 0.04 | 0.15 | 0.12 |
| 113 | 0.04 | 0.14 | 0.14 | 0.04 | 0.15 | 0.12 |
| 114 | 0.04 | 0.14 | 0.14 | 0.04 | 0.15 | 0.12 |
| 115 | 0.04 | 0.15 | 0.15 | 0.04 | 0.15 | 0.12 |
| 116 | 0.04 | 0.15 | 0.15 | 0.04 | 0.16 | 0.12 |
| 117 | 0.04 | 0.15 | 0.15 | 0.04 | 0.16 | 0.13 |
| 118 | 0.04 | 0.15 | 0.15 | 0.04 | 0.16 | 0.13 |
| 119 | 0.04 | 0.15 | 0.15 | 0.04 | 0.17 | 0.14 |
| 120 | 0.04 | 0.16 | 0.15 | 0.04 | 0.17 | 0.14 |
| 121 | 0.04 | 0.16 | 0.16 | 0.04 | 0.17 | 0.14 |
| 122 | 0.05 | 0.16 | 0.16 | 0.05 | 0.17 | 0.14 |
| 123 | 0.05 | 0.16 | 0.16 | 0.05 | 0.17 | 0.14 |
| 124 | 0.05 | 0.16 | 0.16 | 0.05 | 0.17 | 0.14 |
| 125 | 0.05 | 0.16 | 0.16 | 0.05 | 0.18 | 0.15 |
| 126 | 0.05 | 0.17 | 0.17 | 0.05 | 0.18 | 0.15 |
| 127 | 0.05 | 0.17 | 0.17 | 0.05 | 0.18 | 0.15 |
| 128 | 0.05 | 0.17 | 0.17 | 0.05 | 0.18 | 0.16 |
| 129 | 0.05 | 0.17 | 0.17 | 0.05 | 0.19 | 0.16 |
| 130 | 0.05 | 0.18 | 0.17 | 0.05 | 0.19 | 0.17 |
| 131 | 0.05 | 0.18 | 0.17 | 0.05 | 0.19 | 0.17 |
| 132 | 0.05 | 0.18 | 0.18 | 0.05 | 0.19 | 0.17 |
| 133 | 0.05 | 0.18 | 0.18 | 0.05 | 0.19 | 0.17 |
| 134 | 0.05 | 0.18 | 0.18 | 0.05 | 0.2 | 0.17 |
| 135 | 0.05 | 0.19 | 0.18 | 0.05 | 0.2 | 0.17 |
| 136 | 0.05 | 0.19 | 0.18 | 0.05 | 0.2 | 0.18 |
| 137 | 0.05 | 0.19 | 0.19 | 0.06 | 0.2 | 0.18 |
| 138 | 0.05 | 0.2 | 0.19 | 0.06 | 0.21 | 0.19 |
| 139 | 0.05 | 0.2 | 0.19 | 0.06 | 0.21 | 0.2 |
| 140 | 0.05 | 0.2 | 0.19 | 0.06 | 0.22 | 0.2 |
| 141 | 0.05 | 0.2 | 0.2 | 0.06 | 0.22 | 0.21 |
| 142 | 0.06 | 0.2 | 0.2 | 0.06 | 0.22 | 0.21 |
| 143 | 0.06 | 0.21 | 0.2 | 0.06 | 0.22 | 0.21 |
| 144 | 0.06 | 0.21 | 0.2 | 0.06 | 0.22 | 0.21 |
| 145 | 0.06 | 0.21 | 0.2 | 0.06 | 0.23 | 0.21 |
| 146 | 0.06 | 0.21 | 0.2 | 0.07 | 0.23 | 0.22 |
| 147 | 0.06 | 0.21 | 0.21 | 0.07 | 0.23 | 0.22 |
| 148 | 0.06 | 0.21 | 0.21 | 0.07 | 0.23 | 0.22 |
| 149 | 0.06 | 0.22 | 0.21 | 0.07 | 0.24 | 0.23 |
| 150 | 0.06 | 0.22 | 0.21 | 0.07 | 0.24 | 0.23 |
| 151 | 0.06 | 0.22 | 0.22 | 0.07 | 0.24 | 0.23 |
| 152 | 0.06 | 0.22 | 0.22 | 0.07 | 0.25 | 0.24 |
| 153 | 0.06 | 0.22 | 0.22 | 0.07 | 0.25 | 0.24 |
| 154 | 0.06 | 0.23 | 0.22 | 0.07 | 0.25 | 0.24 |
| 155 | 0.06 | 0.23 | 0.22 | 0.07 | 0.25 | 0.25 |
| 156 | 0.06 | 0.23 | 0.22 | 0.07 | 0.26 | 0.25 |
| 157 | 0.06 | 0.23 | 0.23 | 0.07 | 0.26 | 0.25 |
| 158 | 0.06 | 0.23 | 0.23 | 0.07 | 0.27 | 0.26 |
| 159 | 0.07 | 0.24 | 0.23 | 0.08 | 0.27 | 0.26 |
| 160 | 0.07 | 0.24 | 0.23 | 0.08 | 0.27 | 0.27 |
| 161 | 0.07 | 0.24 | 0.24 | 0.08 | 0.28 | 0.28 |
| 162 | 0.07 | 0.25 | 0.25 | 0.08 | 0.28 | 0.29 |
| 163 | 0.07 | 0.25 | 0.25 | 0.08 | 0.29 | 0.29 |
| 164 | 0.07 | 0.25 | 0.25 | 0.08 | 0.29 | 0.29 |
| 165 | 0.07 | 0.25 | 0.25 | 0.08 | 0.29 | 0.3 |
| 166 | 0.07 | 0.25 | 0.25 | 0.09 | 0.3 | 0.3 |
| 167 | 0.07 | 0.25 | 0.25 | 0.09 | 0.3 | 0.31 |
| 168 | 0.07 | 0.26 | 0.26 | 0.09 | 0.3 | 0.31 |
| 169 | 0.08 | 0.26 | 0.26 | 0.09 | 0.31 | 0.32 |
| 170 | 0.08 | 0.26 | 0.26 | 0.09 | 0.31 | 0.32 |
| 171 | 0.08 | 0.27 | 0.27 | 0.09 | 0.31 | 0.32 |
| 172 | 0.08 | 0.27 | 0.27 | 0.09 | 0.32 | 0.33 |
| 173 | 0.08 | 0.27 | 0.28 | 0.09 | 0.32 | 0.34 |
| 174 | 0.08 | 0.28 | 0.28 | 0.1 | 0.33 | 0.35 |
| 175 | 0.08 | 0.28 | 0.28 | 0.1 | 0.34 | 0.35 |
| 176 | 0.08 | 0.28 | 0.28 | 0.1 | 0.34 | 0.37 |
| 177 | 0.08 | 0.28 | 0.29 | 0.1 | 0.35 | 0.38 |
| 178 | 0.08 | 0.28 | 0.29 | 0.1 | 0.35 | 0.38 |
| 179 | 0.08 | 0.29 | 0.29 | 0.1 | 0.35 | 0.38 |
| 180 | 0.08 | 0.29 | 0.29 | 0.1 | 0.36 | 0.39 |
| 181 | 0.08 | 0.29 | 0.3 | 0.1 | 0.36 | 0.39 |
| 182 | 0.09 | 0.29 | 0.3 | 0.11 | 0.36 | 0.39 |
| 183 | 0.09 | 0.29 | 0.3 | 0.11 | 0.37 | 0.4 |
| 184 | 0.09 | 0.3 | 0.3 | 0.11 | 0.37 | 0.4 |
| 185 | 0.09 | 0.3 | 0.31 | 0.11 | 0.37 | 0.41 |
| 186 | 0.09 | 0.3 | 0.31 | 0.11 | 0.38 | 0.41 |
| 187 | 0.09 | 0.31 | 0.31 | 0.11 | 0.38 | 0.42 |
| 188 | 0.09 | 0.31 | 0.31 | 0.11 | 0.39 | 0.42 |
| 189 | 0.09 | 0.31 | 0.32 | 0.11 | 0.39 | 0.43 |
| 190 | 0.09 | 0.31 | 0.32 | 0.12 | 0.39 | 0.43 |
| 191 | 0.09 | 0.32 | 0.32 | 0.12 | 0.4 | 0.44 |
| 192 | 0.09 | 0.32 | 0.32 | 0.12 | 0.4 | 0.44 |
| 193 | 0.09 | 0.32 | 0.32 | 0.12 | 0.41 | 0.45 |
| 194 | 0.09 | 0.32 | 0.33 | 0.12 | 0.41 | 0.45 |
| 195 | 0.1 | 0.33 | 0.33 | 0.13 | 0.42 | 0.46 |
| 196 | 0.1 | 0.33 | 0.34 | 0.13 | 0.42 | 0.48 |
| 197 | 0.1 | 0.34 | 0.35 | 0.13 | 0.43 | 0.49 |
| 198 | 0.1 | 0.34 | 0.35 | 0.13 | 0.44 | 0.51 |
| 199 | 0.1 | 0.34 | 0.35 | 0.14 | 0.45 | 0.51 |
| 200 | 0.1 | 0.34 | 0.35 | 0.14 | 0.45 | 0.52 |
| 201 | 0.1 | 0.35 | 0.35 | 0.14 | 0.46 | 0.53 |
| 202 | 0.1 | 0.35 | 0.36 | 0.14 | 0.46 | 0.54 |
| 203 | 0.11 | 0.35 | 0.36 | 0.15 | 0.47 | 0.55 |
| 204 | 0.11 | 0.36 | 0.37 | 0.15 | 0.48 | 0.56 |
| 205 | 0.11 | 0.36 | 0.37 | 0.15 | 0.48 | 0.57 |
| 206 | 0.11 | 0.37 | 0.37 | 0.16 | 0.49 | 0.57 |
| 207 | 0.11 | 0.37 | 0.38 | 0.16 | 0.5 | 0.58 |
| 208 | 0.11 | 0.37 | 0.38 | 0.16 | 0.5 | 0.59 |
| 209 | 0.11 | 0.37 | 0.38 | 0.17 | 0.51 | 0.61 |
| 210 | 0.11 | 0.38 | 0.38 | 0.18 | 0.52 | 0.62 |
| 211 | 0.11 | 0.38 | 0.39 | 0.18 | 0.53 | 0.63 |
| 212 | 0.11 | 0.38 | 0.39 | 0.19 | 0.54 | 0.64 |
| 213 | 0.11 | 0.39 | 0.4 | 0.2 | 0.54 | 0.65 |
| 214 | 0.11 | 0.39 | 0.41 | 0.2 | 0.55 | 0.66 |
| 215 | 0.12 | 0.4 | 0.41 | 0.2 | 0.55 | 0.66 |
| 216 | 0.12 | 0.4 | 0.42 | 0.21 | 0.56 | 0.67 |
| 217 | 0.12 | 0.4 | 0.42 | 0.21 | 0.57 | 0.68 |
| 218 | 0.12 | 0.41 | 0.42 | 0.21 | 0.57 | 0.68 |
| 219 | 0.12 | 0.41 | 0.42 | 0.22 | 0.58 | 0.69 |
| 220 | 0.12 | 0.41 | 0.42 | 0.22 | 0.58 | 0.7 |
| 221 | 0.12 | 0.41 | 0.43 | 0.23 | 0.59 | 0.71 |
| 222 | 0.12 | 0.42 | 0.43 | 0.24 | 0.6 | 0.72 |
| 223 | 0.12 | 0.42 | 0.43 | 0.24 | 0.61 | 0.73 |
| 224 | 0.12 | 0.42 | 0.44 | 0.25 | 0.62 | 0.74 |
| 225 | 0.12 | 0.43 | 0.44 | 0.25 | 0.62 | 0.75 |
| 226 | 0.12 | 0.43 | 0.44 | 0.26 | 0.63 | 0.77 |
| 227 | 0.12 | 0.43 | 0.45 | 0.27 | 0.65 | 0.78 |
| 228 | 0.13 | 0.43 | 0.45 | 0.28 | 0.65 | 0.79 |
| 229 | 0.13 | 0.44 | 0.45 | 0.29 | 0.66 | 0.8 |
| 230 | 0.13 | 0.44 | 0.46 | 0.3 | 0.67 | 0.82 |
| 231 | 0.13 | 0.45 | 0.46 | 0.3 | 0.68 | 0.82 |
| 232 | 0.13 | 0.45 | 0.47 | 0.31 | 0.69 | 0.84 |
| 233 | 0.13 | 0.45 | 0.47 | 0.32 | 0.7 | 0.85 |
| 234 | 0.13 | 0.45 | 0.47 | 0.33 | 0.71 | 0.87 |
| 235 | 0.13 | 0.46 | 0.48 | 0.34 | 0.73 | 0.89 |
| 236 | 0.13 | 0.46 | 0.48 | 0.35 | 0.73 | 0.9 |
| 237 | 0.14 | 0.47 | 0.48 | 0.36 | 0.75 | 0.92 |
| 238 | 0.14 | 0.47 | 0.49 | 0.37 | 0.76 | 0.93 |
| 239 | 0.14 | 0.47 | 0.49 | 0.39 | 0.78 | 0.96 |
| 240 | 0.14 | 0.48 | 0.49 | 0.4 | 0.79 | 0.97 |
| 241 | 0.14 | 0.48 | 0.5 | 0.41 | 0.81 | 0.99 |
| 242 | 0.14 | 0.48 | 0.5 | 0.42 | 0.82 | 1.01 |
| 243 | 0.15 | 0.49 | 0.51 | 0.44 | 0.83 | 1.03 |
| 244 | 0.15 | 0.49 | 0.51 | 0.45 | 0.85 | 1.05 |
| 245 | 0.15 | 0.49 | 0.52 | 0.46 | 0.86 | 1.07 |
| 246 | 0.15 | 0.5 | 0.52 | 0.48 | 0.88 | 1.09 |
| 247 | 0.15 | 0.5 | 0.52 | 0.49 | 0.89 | 1.1 |
| 248 | 0.16 | 0.51 | 0.53 | 0.5 | 0.9 | 1.12 |
| 249 | 0.16 | 0.51 | 0.53 | 0.51 | 0.92 | 1.14 |
| 250 | 0.15 | 0.48 | 0.5 | 0.52 | 0.93 | 1.16 |
| 251 | 0.15 | 0.64 | 0.49 | 0.56 | 0.97 | 1.2 |

Sequencing the samples with an Illumina MiSeq yielded 251 bp paired-end data, meaning each sample had a file containing forward sequences and another file containing reverse sequences. Quality is reported here as Expected Error, the sum of all the error probabilities (based on phred Q scores) through the given bp position.

## Supplementary Table 6: Bacteria enrichment following each diet compared to baseline in participants at risk of CVD.

| **Comparison** | **Diet** | **LDA Score** | **RA (%)** | **P Value** |
| --- | --- | --- | --- | --- |
| ***LSD vs. Baseline*** | | | | |
| D1_Bacteroidetes D2_Bacteroidia D3_Bacteroidales D4_Rikenellaceae | LSD | 3.46 | 0.63 | 0.017 |
| D1_Bacteroidetes D2_Bacteroidia D3_Bacteroidales D4_Rikenellaceae D5_Alistipes | LSD | 3.46 | 0.63 | 0.019 |
| D1_Firmicutes D2_Clostridia D3_Clostridiales D4_Lachnospiraceae._ | LSD | 3.33 | 0.93 | 0.023 |
| D1_Firmicutes D2_Clostridia D3_Clostridiales D4_Lachnospiraceae._._ | LSD | 3.33 | 0.93 | 0.023 |
| D1_Firmicutes D2_Bacilli D3_Lactobacillales D4_Streptococcaceae D5_Streptococcus D6_Streptococcus_salivarius_subsp_thermophilus | LSD | 3.23 | 0.43 | 0.007 |
| D1_Firmicutes D2_Bacilli D3_Lactobacillales D4_Streptococcaceae D5_Streptococcus | LSD | 3.23 | 0.48 | 0.008 |
| D1_Firmicutes D2_Bacilli D3_Lactobacillales D4_Streptococcaceae | LSD | 3.20 | 0.50 | 0.008 |
| D1_Bacteroidetes D2_Bacteroidia D3_Bacteroidales D4_Rikenellaceae D5_Alistipes._ | LSD | 3.20 | 0.42 | 0.041 |
| D1_Firmicutes D2_Bacilli D3_Lactobacillales | LSD | 3.08 | 0.51 | 0.020 |
| D1_Firmicutes D2_Bacilli | LSD | 3.08 | 0.51 | 0.020 |
| D1_Firmicutes D2_Clostridia D3_Clostridiales D4_Ruminococcaceae D5_Ruminococcaceae_UCG_005 | LSD | 3.06 | 0.12 | 0.006 |
| D1_Firmicutes D2_Clostridia D3_Clostridiales D4_Ruminococcaceae D5_uncultured | LSD | 2.96 | 0.06 | 0.024 |
| D1_Bacteroidetes D2_Bacteroidia D3_Bacteroidales D4_Rikenellaceae D5_Alistipes D6_uncultured_organism | LSD | 2.91 | 0 | 0.029 |
| D1_Tenericutes D2_Mollicutes D3_Mollicutes_RF39 D4_gut_metagenome | LSD | 2.88 | 0 | 0.033 |
| D1_Tenericutes D2_Mollicutes D3_Mollicutes_RF39 D4_gut_metagenome D5_gut_metagenome D6_gut_metagenome | LSD | 2.85 | 0 | 0.033 |
| D1_Tenericutes D2_Mollicutes D3_Mollicutes_RF39 D4_gut_metagenome D5_gut_metagenome | LSD | 2.85 | 0 | 0.033 |
| D1_Actinobacteria D2_Coriobacteriia D3_Coriobacteriales D4_Coriobacteriales_Incertae_Sedis | LSD | 2.83 | 0.05 | 0.020 |
| D1_Actinobacteria D2_Coriobacteriia D3_Coriobacteriales D4_Coriobacteriales_Incertae_Sedis D5_uncultured | LSD | 2.82 | 0.04 | 0.024 |
| D1_Firmicutes D2_Clostridia D3_Clostridiales D4_Ruminococcaceae D5_Ruminococcaceae_UCG_005._ | LSD | 2.58 | 0 | 0.037 |
| D1_Firmicutes D2_Clostridia D3_Clostridiales D4_Ruminococcaceae D5_Ruminococcaceae_UCG_005 D6_human_gut_metagenome | LSD | 2.57 | 0 | 0.002 |
| D1_Proteobacteria D2_Deltaproteobacteria D3_Desulfovibrionales D4_Desulfovibrionaceae D5_Desulfovibrio | LSD | 2.45 | 0 | 0.037 |
| D1_Firmicutes D2_Clostridia D3_Clostridiales D4_Ruminococcaceae D5_uncultured._ | LSD | 2.25 | 0 | 0.002 |
| D1_Firmicutes D2_Clostridia D3_Clostridiales D4_Clostridiales_vadinBB60_group | LSD | 2.11 | 0 | 0.003 |
| D1_Bacteroidetes D2_Bacteroidia D3_Bacteroidales D4_Marinifilaceae D5_Odoribacter._ | LSD | 2.08 | 0 | 0.018 |
| D1_Firmicutes D2_Clostridia D3_Clostridiales D4_Lachnospiraceae D5_Ruminococcus_gauvreauii_group | B | 3.61 | 0.51 | <0.001 |
| D1_Firmicutes D2_Clostridia D3_Clostridiales D4_Lachnospiraceae D5_Ruminococcus_gauvreauii_group._ | B | 3.61 | 0.51 | <0.001 |
| D1_Proteobacteria D2_Gammaproteobacteria | B | 3.40 | 0.07 | 0.043 |
| D1_Proteobacteria | B | 3.38 | 0.13 | 0.032 |
| D1_Firmicutes D2_Clostridia D3_Clostridiales D4_Ruminococcaceae D5_Ruminococcaceae_UCG_013 | B | 3.05 | 0.50 | 0.007 |
| D1_Firmicutes D2_Clostridia D3_Clostridiales D4_Ruminococcaceae D5_Ruminococcaceae_UCG_013 D6_uncultured_organism | B | 2.98 | 0.35 | 0.023 |
| D1_Firmicutes D2_Negativicutes D3_Selenomonadales D4_Veillonellaceae D5_Dialister | B | 2.83 | 0.01 | 0.040 |
| D1_Firmicutes D2_Negativicutes D3_Selenomonadales D4_Veillonellaceae D5_Dialister._ | B | 2.83 | 0.01 | 0.040 |
| D1_Bacteroidetes D2_Bacteroidia D3_Bacteroidales D4_Muribaculaceae | B | 2.72 | 0 | 0.036 |
| D1_Firmicutes D2_Clostridia D3_Clostridiales D4_Ruminococcaceae D5_Ruminococcaceae_UCG_013 D6_gut_metagenome | B | 2.59 | 0 | 0.012 |
| D1_Firmicutes D2_Clostridia D3_Clostridiales D4_Ruminococcaceae D5_Ruminiclostridium_5 D6_gut_metagenome | B | 2.49 | 0.03 | 0.013 |
| ***MSD vs. Baseline*** | | | | |
| D1_Firmicutes D2_Clostridia D3_Clostridiales D4_Ruminococcaceae | MSD | 4.36 | 22.14 | 0.004 |
| D1_Bacteroidetes D2_Bacteroidia D3_Bacteroidales D4_Bacteroidaceae D5_Bacteroides._ | MSD | 4.06 | 2.07 | 0.044 |
| D1_Firmicutes D2_Clostridia D3_Clostridiales D4_Ruminococcaceae D5_Ruminococcus_2 | MSD | 3.99 | 2.10 | 0.003 |
| D1_Firmicutes D2_Clostridia D3_Clostridiales D4_Ruminococcaceae D5_Ruminococcus_2 D6_uncultured_bacterium | MSD | 3.99 | 2.10 | 0.003 |
| D1_Firmicutes D2_Clostridia D3_Clostridiales D4_Ruminococcaceae D5_Subdoligranulum | MSD | 3.74 | 3.73 | 0.044 |
| D1_Firmicutes D2_Clostridia D3_Clostridiales D4_Ruminococcaceae D5_Subdoligranulum._ | MSD | 3.74 | 3.73 | 0.048 |
| D1_Firmicutes D2_Bacilli D3_Lactobacillales D4_Streptococcaceae D5_Streptococcus | MSD | 3.49 | 0.40 | 0.003 |
| D1_Firmicutes D2_Bacilli D3_Lactobacillales D4_Streptococcaceae D5_Streptococcus D6_Streptococcus_salivarius_subsp_thermophilus | MSD | 3.49 | 0.37 | 0.001 |
| D1_Firmicutes D2_Bacilli D3_Lactobacillales D4_Streptococcaceae | MSD | 3.47 | 0.41 | 0.005 |
| D1_Bacteroidetes D2_Bacteroidia D3_Bacteroidales D4_Rikenellaceae | MSD | 3.44 | 0.55 | <0.001 |
| D1_Bacteroidetes D2_Bacteroidia D3_Bacteroidales D4_Rikenellaceae D5_Alistipes | MSD | 3.43 | 0.55 | <0.001 |
| D1_Firmicutes D2_Bacilli D3_Lactobacillales | MSD | 3.36 | 0.44 | 0.005 |
| D1_Firmicutes D2_Bacilli | MSD | 3.36 | 0.44 | 0.006 |
| D1_Firmicutes D2_Clostridia D3_Clostridiales D4_Ruminococcaceae D5_Ruminococcaceae_UCG_002 | MSD | 3.34 | 0.36 | 0.004 |
| D1_Firmicutes D2_Clostridia D3_Clostridiales D4_Ruminococcaceae D5_Ruminococcus_1 | MSD | 3.26 | 1.12 | 0.043 |
| D1_Bacteroidetes D2_Bacteroidia D3_Bacteroidales D4_Rikenellaceae D5_Alistipes._ | MSD | 3.26 | 0.38 | 0.001 |
| D1_Firmicutes D2_Clostridia D3_Clostridiales D4_Lachnospiraceae D5_Roseburia D6_uncultured_Roseburia_sp_ | MSD | 3.21 | 0 | 0.007 |
| D1_Firmicutes D2_Clostridia. D3_Clostridiales D4_Ruminococcaceae D5_Ruminiclostridium_6 | MSD | 3.10 | 0.03 | 0.011 |
| D1_Bacteroidetes D2_Bacteroidia D3_Bacteroidales D4_Prevotellaceae D5_Paraprevotella | MSD | 3.02 | 0 | 0.014 |
| D1_Firmicutes D2_Clostridia D3_Clostridiales D4_Ruminococcaceae D5_Ruminococcaceae_UCG_002._ | MSD | 2.97 | 0.15 | 0.017 |
| D1_Firmicutes D2_Clostridia D3_Clostridiales D4_Ruminococcaceae D5_Ruminococcaceae_UCG_002 D6_uncultured_rumen_bacterium | MSD | 2.96 | 0 | 0.002 |
| D1_Firmicutes D2_Clostridia D3_Clostridiales D4_Ruminococcaceae D5_Ruminococcus_1 D6_uncultured_organism | MSD | 2.93 | 0.14 | 0.043 |
| D1_Firmicutes D2_Clostridia D3_Clostridiales D4_Ruminococcaceae D5_Ruminococcus_1 D6_metagenome | MSD | 2.89 | 0 | 0.021 |
| D1_Firmicutes D2_Clostridia D3_Clostridiales D4_Ruminococcaceae D5_uncultured | MSD | 2.83 | 0.10 | 0.008 |
| D1_Firmicutes D2_Clostridia D3_Clostridiales D4_Ruminococcaceae D5_Ruminococcaceae_UCG_005 | MSD | 2.82 | 0.09 | 0.011 |
| D1_Firmicutes D2_Clostridia D3_Clostridiales D4_Christensenellaceae D5_Christensenellaceae_R_7_group D6_uncultured_organism | MSD | 2.78 | 0 | 0.018 |
| D1_Tenericutes D2_Mollicutes | MSD | 2.75 | 0 | 0.014 |
| D1_Tenericutes | MSD | 2.72 | 0 | 0.014 |
| D1_Bacteroidetes D2_Bacteroidia D3_Bacteroidales D4_Prevotellaceae D5_Paraprevotella._ | MSD | 2.66 | 0 | 0.032 |
| D1_Bacteroidetes D2_Bacteroidia D3_Bacteroidales D4_Rikenellaceae D5_Alistipes D6_uncultured_organism | MSD | 2.65 | 0 | 0.026 |
| D1_Firmicutes D2_Clostridia D3_Clostridiales D4_Ruminococcaceae D5_Ruminiclostridium_6._ | MSD | 2.50 | 0 | 0.045 |
| D1_Firmicutes D2_Clostridia D3_Clostridiales D4_Ruminococcaceae D5_Ruminococcaceae_UCG_005._ | MSD | 2.49 | 0 | 0.048 |
| D1_Firmicutes D2_Clostridia D3_Clostridiales D4_Ruminococcaceae D5_Oscillibacter D6_uncultured_organism | MSD | 2.39 | 0.03 | 0.031 |
| D1_Firmicutes D2_Clostridia D3_Clostridiales D4_Ruminococcaceae D5_uncultured._ | MSD | 2.38 | 0 | 0.040 |
| D1_Firmicutes D2_Clostridia D3_Clostridiales D4_Ruminococcaceae D5_Ruminococcaceae_UCG_010 | MSD | 2.35 | 0 | 0.041 |
| D1_Firmicutes D2_Clostridia D3_Clostridiales D4_Ruminococcaceae D5_Ruminococcaceae_UCG_003 D6_uncultured_organism | MSD | 2.33 | 0.02 | 0.005 |
| D1_Firmicutes D2_Clostridia D3_Clostridiales D4_Ruminococcaceae D5_Ruminococcaceae_UCG_003 | MSD | 2.31 | 0.02 | 0.011 |
| D1_Bacteroidetes D2_Bacteroidia D3_Bacteroidales D4_Rikenellaceae D5_Alistipes D6_Alistipes_obesi | MSD | 2.31 | 0 | 0.019 |
| D1_Proteobacteria D2_Deltaproteobacteria D3_Desulfovibrionales | MSD | 2.25 | 0.02 | 0.009 |
| D1_Proteobacteria D2_Deltaproteobacteria | MSD | 2.25 | 0.02 | 0.009 |
| D1_Firmicutes D2_Clostridia D3_Clostridiales D4_Ruminococcaceae D5_Intestinimonas | MSD | 2.24 | 0 | 0.044 |
| D1_Proteobacteria D2_Deltaproteobacteria D3_Desulfovibrionales D4_Desulfovibrionaceae | MSD | 2.24 | 0.02 | 0.009 |
| D1_Proteobacteria D2_Deltaproteobacteria D3_Desulfovibrionales D4_Desulfovibrionaceae D5_Desulfovibrio | MSD | 2.15 | 0 | 0.009 |
| D1_Firmicutes D2_Clostridia D3_Clostridiales D4_Clostridiales_vadinBB60_group | MSD | 2.13 | 0 | 0.002 |
| D1_Firmicutes D2_Clostridia D3_Clostridiales D4_Ruminococcaceae D5_Ruminococcaceae_UCG_005 D6_human_gut_metagenome | MSD | 2.09 | 0 | 0.003 |
| D1_Firmicutes D2_Clostridia D3_Clostridiales. D4_Lachnospiraceae | B | 4.54 | 49.42 | 0.035 |
| D1_Actinobacteria | B | 3.98 | 3.48 | 0.006 |
| D1_Actinobacteria D2_Actinobacteria D3_Bifidobacteriales D4_Bifidobacteriaceae D5_Bifidobacterium | B | 3.93 | 1.60 | 0.022 |
| D1_Actinobacteria D2_Actinobacteria D3_Bifidobacteriales | B | 3.93 | 1.60 | 0.022 |
| D1_Actinobacteria D2_Actinobacteria D3_Bifidobacteriales D4_Bifidobacteriaceae | B | 3.93 | 1.60 | 0.022 |
| D1_Actinobacteria D2_Actinobacteria | B | 3.93 | 1.60 | 0.026 |
| D1_Actinobacteria D2_Actinobacteria D3_Bifidobacteriales D4_Bifidobacteriaceae D5_Bifidobacterium._ | B | 3.92 | 1.60 | 0.019 |
| D1_Firmicutes D2_Clostridia D3_Clostridiales D4_Lachnospiraceae D5_Eubacterium_hallii_group | B | 3.86 | 3.59 | 0.002 |
| D1_Firmicutes D2_Clostridia D3_Clostridiales D4_Lachnospiraceae D5_Eubacterium_hallii_group._ | B | 3.86 | 3.59 | 0.002 |
| D1_Firmicutes D2_Clostridia D3_Clostridiales D4_Lachnospiraceae D5_Ruminococcus_torques_group | B | 3.77 | 1.42 | <0.001 |
| D1_Firmicutes D2_Clostridia D3_Clostridiales D4_Lachnospiraceae D5_Ruminococcus_torques_group._ | B | 3.76 | 1.07 | <0.001 |
| D1_Firmicutes D2_Clostridia D3_Clostridiales D4_Lachnospiraceae D5_Dorea | B | 3.48 | 2.59 | 0.040 |
| D1_Firmicutes D2_Clostridia D3_Clostridiales D4_Lachnospiraceae D5_Dorea._ | B | 3.48 | 2.59 | 0.040 |
| D1_Firmicutes D2_Clostridia D3_Clostridiales D4_Lachnospiraceae D5_Ruminococcus_gauvreauii_group | B | 3.45 | 0.51 | 0.004 |
| D1_Firmicutes D2_Clostridia D3_Clostridiales D4_Lachnospiraceae D5_Ruminococcus_gauvreauii_group._ | B | 3.44 | 0.51 | 0.004 |
| D1_Firmicutes D2_Clostridia D3_Clostridiales D4_Lachnospiraceae D5_Coprococcus_3._ | B | 3.30 | 0.99 | 0.013 |
| D1_Firmicutes D2_Clostridia D3_Clostridiales D4_Lachnospiraceae D5_Coprococcus_3 | B | 3.29 | 0.99 | 0.011 |
| D1_Firmicutes D2_Clostridia D3_Clostridiales D4_Ruminococcaceae D5_Ruminiclostridium_5 | B | 3.18 | 0.30 | 0.009 |
| D1_Firmicutes D2_Clostridia D3_Clostridiales D4_Ruminococcaceae D5_Ruminiclostridium_5._ | B | 3.11 | 0.16 | 0.011 |
| D1_Firmicutes D2_Clostridia D3_Clostridiales D4_Ruminococcaceae D5_Ruminococcaceae_UCG_013 | B | 3.03 | 0.50 | 0.012 |
| D1_Firmicutes D2_Clostridia D3_Clostridiales D4_Ruminococcaceae D5_Ruminococcaceae_UCG_013 D6_uncultured_organism | B | 2.98 | 0.35 | 0.020 |
| D1_Firmicutes D2_Clostridia D3_Clostridiales D4_Ruminococcaceae D5_Ruminiclostridium_5 D6_gut_metagenome | B | 2.50 | 0.03 | 0.042 |
| D1_Firmicutes D2_Clostridia D3_Clostridiales D4_Lachnospiraceae D5_Eubacterium_ventriosum_group D6_uncultured_bacterium | B | 2.16 | 0 | 0.036 |
| ***HSD vs. Baseline*** | | | | |
| D1_Firmicutes D2_Clostridia D3_Clostridiales D4_Ruminococcaceae | HSD | 4.62 | 24.90 | <0.001 |
| D1_Firmicutes D2_Clostridia D3_Clostridiales D4_Ruminococcaceae D5_Faecalibacterium | HSD | 4.25 | 7.63 | 0.002 |
| D1_Firmicutes D2_Clostridia D3_Clostridiales D4_Ruminococcaceae D5_Faecalibacterium._ | HSD | 4.24 | 6.86 | 0.002 |
| D1_Firmicutes D2_Clostridia D3_Clostridiales D4_Ruminococcaceae D5_Ruminococcus_2 | HSD | 4.11 | 3.34 | <0.001 |
| D1_Firmicutes D2_Clostridia D3_Clostridiales D4_Ruminococcaceae D5_Ruminococcus_2 D6_uncultured_bacterium | HSD | 4.08 | 3.19 | <0.001 |
| D1_Firmicutes D2_Clostridia D3_Clostridiales D4_Lachnospiraceae D5_Agathobacter._ | HSD | 3.98 | 4.55 | 0.032 |
| D1_Firmicutes D2_Clostridia D3_Clostridiales D4_Ruminococcaceae D5_Subdoligranulum | HSD | 3.86 | 4.31 | 0.034 |
| D1_Firmicutes D2_Clostridia D3_Clostridiales D4_Ruminococcaceae D5_Subdoligranulum._ | HSD | 3.86 | 4.31 | 0.034 |
| D1_Bacteroidetes D2_Bacteroidia D3_Bacteroidales D4_Rikenellaceae | HSD | 3.44 | 0.65 | 0.008 |
| D1_Bacteroidetes D2_Bacteroidia D3_Bacteroidales D4_Rikenellaceae D5_Alistipes | HSD | 3.42 | 0.65 | 0.011 |
| D1_Firmicutes D2_Clostridia D3_Clostridiales D4_Ruminococcaceae D5_Ruminococcaceae_UCG_002 | HSD | 3.41 | 0.45 | 0.001 |
| D1_Firmicutes D2_Clostridia D3_Clostridiales D4_Lachnospiraceae._ | HSD | 3.32 | 1.28 | 0.004 |
| D1_Firmicutes D2_Clostridia D3_Clostridiales D4_Lachnospiraceae._._ | HSD | 3.32 | 1.28 | 0.004 |
| D1_Bacteroidetes D2_Bacteroidia D3_Bacteroidales D4_Rikenellaceae D5_Alistipes._ | HSD | 3.26 | 0.36 | 0.014 |
| D1_Firmicutes D2_Clostridia D3_Clostridiales D4_Ruminococcaceae D5_Ruminococcaceae_UCG_002._ | HSD | 3.19 | 0.14 | 0.002 |
| D1_Firmicutes D2_Clostridia D3_Clostridiales D4_Ruminococcaceae D5_Ruminiclostridium_6 | HSD | 2.99 | 0.03 | 0.005 |
| D1_Firmicutes D2_Clostridia D3_Clostridiales D4_Ruminococcaceae D5_uncultured | HSD | 2.98 | 0.08 | 0.018 |
| D1_Firmicutes D2_Clostridia D3_Clostridiales D4_Lachnospiraceae D5_Roseburia D6_uncultured_Roseburia_sp_ | HSD | 2.96 | 0 | 0.033 |
| D1_Firmicutes D2_Clostridia D3_Clostridiales D4_Ruminococcaceae D5_Ruminococcaceae_UCG_005 | HSD | 2.89 | 0.11 | 0.006 |
| D1_Firmicutes D2_Clostridia D3_Clostridiales D4_Ruminococcaceae D5_Ruminococcaceae_UCG_002 D6_uncultured_rumen_bacterium | HSD | 2.88 | 0 | 0.004 |
| D1_Firmicutes D2_Clostridia D3_Clostridiales D4_Ruminococcaceae D5_Ruminococcus_1 D6_metagenome | HSD | 2.72 | 0 | 0.037 |
| D1_Firmicutes D2_Clostridia D3_Clostridiales D4_Lachnospiraceae D5_Eubacterium_xylanophilum_group._ | HSD | 2.54 | 0.05 | 0.032 |
| D1_Firmicutes D2_Clostridia D3_Clostridiales D4_Ruminococcaceae D5_Ruminococcaceae_UCG_005._ | HSD | 2.41 | 0 | 0.005 |
| D1_Firmicutes D2_Clostridia D3_Clostridiales D4_Ruminococcaceae D5_Ruminiclostridium_6._ | HSD | 2.37 | 0 | 0.018 |
| D1_Firmicutes D2_Clostridia D3_Clostridiales D4_Ruminococcaceae D5_UBA1819 | HSD | 2.24 | 0.04 | 0.045 |
| D1_Firmicutes D2_Clostridia D3_Clostridiales D4_Ruminococcaceae D5_Ruminococcaceae_UCG_005 D6_human_gut_metagenome | HSD | 2.17 | 0 | 0.025 |
| D1_Firmicutes D2_Clostridia D3_Clostridiales D4_Ruminococcaceae D5_uncultured D6_Clostridium_phoceensis | HSD | 2.07 | 0 | 0.016 |
| D1_Proteobacteria D2_Alphaproteobacteria D3_Rhodospirillales D4_uncultured | HSD | 2.01 | 0 | 0.030 |
| D1_Firmicutes D2_Clostridia D3_Clostridiales D4_Lachnospiraceae | B | 4.66 | 49.42 | 0.002 |
| D1_Firmicutes D2_Clostridia D3_Clostridiales D4_Lachnospiraceae D5_Blautia | B | 4.49 | 21.63 | 0.001 |
| D1_Firmicutes D2_Clostridia D3_Clostridiales D4_Lachnospiraceae D5_Blautia._ | B | 4.48 | 20.94 | 0.002 |
| D1_Firmicutes D2_Clostridia D3_Clostridiales D4_Lachnospiraceae D5_Eubacterium_hallii_group | B | 3.92 | 3.59 | 0.002 |
| D1_Firmicutes D2_Clostridia D3_Clostridiales D4_Lachnospiraceae D5_Eubacterium_hallii_group._ | B | 3.92 | 3.59 | 0.003 |
| D1_Firmicutes D2_Clostridia D3_Clostridiales D4_Lachnospiraceae D5_Ruminococcus_torques_group | B | 3.85 | 1.42 | 0.001 |
| D1_Firmicutes D2_Clostridia D3_Clostridiales D4_Lachnospiraceae D5_Ruminococcus_torques_group._ | B | 3.81 | 1.07 | 0.006 |
| D1_Firmicutes D2_Clostridia D3_Clostridiales D4_Lachnospiraceae D5_Dorea | B | 3.66 | 2.59 | 0.002 |
| D1_Firmicutes D2_Clostridia D3_Clostridiales D4_Lachnospiraceae D5_Dorea._ | B | 3.66 | 2.59 | 0.002 |
| D1_Firmicutes D2_Clostridia D3_Clostridiales D4_Lachnospiraceae D5_Fusicatenibacter | B | 3.47 | 2.04 | 0.042 |
| D1_Firmicutes D2_Clostridia D3_Clostridiales D4_Lachnospiraceae D5_Fusicatenibacter._ | B | 3.47 | 2.04 | 0.042 |
| D1_Actinobacteria D2_Coriobacteriia D3_Coriobacteriales D4_Coriobacteriaceae | B | 3.43 | 0.57 | 0.005 |
| D1_Actinobacteria D2_Coriobacteriia D3_Coriobacteriales D4_Coriobacteriaceae D5_Collinsella | B | 3.43 | 0.57 | 0.005 |
| D1_Actinobacteria D2_Coriobacteriia D3_Coriobacteriales D4_Coriobacteriaceae D5_Collinsella._ | B | 3.43 | 0.57 | 0.005 |
| D1_Firmicutes D2_Clostridia D3_Clostridiales D4_Lachnospiraceae D5_Ruminococcus_gauvreauii_group | B | 3.42 | 0.51 | 0.002 |
| D1_Firmicutes D2_Clostridia D3_Clostridiales D4_Lachnospiraceae D5_Ruminococcus_gauvreauii_group._ | B | 3.42 | 0.51 | 0.001 |
| D1_Actinobacteria D2_Coriobacteriia | B | 3.41 | 1.12 | 0.015 |
| D1_Actinobacteria D2_Coriobacteriia D3_Coriobacteriales | B | 3.41 | 1.12 | 0.015 |
| D1_Firmicutes D2_Clostridia D3_Clostridiales D4_Lachnospiraceae D5_Coprococcus_3 | B | 3.35 | 0.99 | 0.012 |
| D1_Firmicutes D2_Clostridia D3_Clostridiales D4_Lachnospiraceae D5_Coprococcus_3._ | B | 3.33 | 0.99 | 0.012 |
| D1_Firmicutes D2_Clostridia D3_Clostridiales D4_Ruminococcaceae D5_Ruminiclostridium_5 | B | 3.12 | 0.30 | 0.016 |
| D1_Firmicutes D2_Clostridia D3_Clostridiales D4_Lachnospiraceae D5_Ruminococcus_gnavus_group | B | 3.10 | 0 | 0.009 |
| D1_Firmicutes D2_Clostridia D3_Clostridiales D4_Lachnospiraceae D5_Ruminococcus_gnavus_group._ | B | 3.08 | 0 | 0.009 |
| D1_Firmicutes D2_Clostridia D3_Clostridiales D4_Ruminococcaceae D5_Ruminiclostridium_5._ | B | 3.06 | 0.16 | 0.023 |
| D1_Firmicutes D2_Clostridia D3_Clostridiales D4_Lachnospiraceae D5_Lachnoclostridium._ | B | 2.81 | 0.08 | 0.029 |
| D1_Firmicutes D2_Clostridia D3_Clostridiales D4_Lachnospiraceae D5_Lachnoclostridium D6_Clostridium_scindens | B | 2.34 | 0 | 0.038 |
| D1_Firmicutes D2_Clostridia D3_Clostridiales D4_Lachnospiraceae D5_Blautia D6_Blautia_hydrogenotrophica | B | 2.27 | 0 | 0.040 |
| D1_Actinobacteria D2_Actinobacteria D3_Bifidobacteriales D4_Bifidobacteriaceae D5_Bifidobacterium D6_Bifidobacterium_bifidum | B | 2.26 | 0 | 0.044 |
| D1_Firmicutes D2_Bacilli D3_Lactobacillales D4_Streptococcaceae D5_Lactococcus._ | B | 2.25 | 0 | 0.032 |
| D1_Firmicutes D2_Erysipelotrichia D3_Erysipelotrichales D4_Erysipelotrichaceae D5_Erysipelatoclostridium | B | 2.23 | 0.02 | 0.010 |
| D1_Firmicutes D2_Bacilli D3_Lactobacillales D4_Streptococcaceae D5_Lactococcus | B | 2.20 | 0 | 0.032 |

LDA scores quantify the strength of enrichment within each respective categorical group. The p values were derived from Wilcoxon signed-rank tests.

B, Baseline; LDA, Linear discriminant analysis; LSD, low spice diet; MSD, moderate spice diet; HSD, high spice diet; RA, relative abundance presented as the median percent.

D1: Phylum; D2: Class; D3: Order; D4: Family; D5: Genus; D6: Species

## Supplementary Table 7: Predictive functional pathway enrichment following the spice-containing diets compared to baseline in participants at risk of CVD.

| **Comparison** | **Diet** | **LDA Score** | **P Value** |
| --- | --- | --- | --- |
| ***LSD vs. Baseline*** |  |  |  |
| Glycan biosynthesis and metabolism | LSD | 2.90 | 0.035 |
| Other glycan degradation PATH ko00511_ | LSD | 2.43 | 0.021 |
| Lipopolysaccharide biosynthesis PATH ko00540 | LSD | 2.42 | 0.049 |
| Lysosome PATH ko04142 | LSD | 2.14 | 0.044 |
| Glycosaminoglycan degradation PATH ko00531 | LSD | 2.03 | 0.033 |
| Phenylalanine metabolism PATH ko00360 | LSD | 2.03 | 0.016 |
| Membrane transport | B | 3.17 | 0.042 |
| Quorum sensing PATH ko02024_ | B | 2.67 | 0.019 |
| Cellular community prokaryotes | B | 2.54 | 0.016 |
| Pentose phosphate pathway PATH ko00030 | B | 2.14 | 0.035 |
| Lysine biosynthesis PATH ko00300 | B | 2.04 | 0.016 |
| ***MSD vs. Baseline*** |  |  |  |
| Glycan biosynthesis and metabolism | MSD | 2.87 | 0.035 |
| Other glycan degradation PATH ko00511 | MSD | 2.44 | 0.026 |
| Amino sugar and nucleotide sugar metabolism PATH ko00520 | MSD | 2.44 | 0.001 |
| Lysosome PATH ko04142 | MSD | 2.20 | 0.009 |
| Glycosaminoglycan degradation PATH ko00531 | MSD | 2.11 | 0.006 |
| Histidine metabolism PATH ko00340 | MSD | 2.11 | 0.003 |
| Xenobiotics biodegradation and metabolism | MSD | 2.02 | 0.018 |
| Quorum sensing PATH ko02024 | B | 2.68 | 0.031 |
| Cellular community prokaryotes | B | 2.61 | 0.034 |
| Metabolism of cofactors and vitamins | B | 2.58 | 0.028 |
| Porphyrin and chlorophyll metabolism PATH ko00860 | B | 2.49 | 0.018 |
| Pentose phosphate pathway PATH ko00030 | B | 2.19 | 0.025 |
| Folate biosynthesis PATH ko00790 | B | 2.09 | 0.009 |
| Lysine biosynthesis PATH ko00300 | B | 2.07 | 0.015 |
| ***HSD vs. Baseline*** |  |  |  |
| Genetic Information Processing | HSD | 3.00 | 0.005 |
| Glycan biosynthesis and metabolism | HSD | 2.82 | 0.028 |
| Flagellar assembly PATH ko02040 | HSD | 2.76 | 0.008 |
| Replication and repair | HSD | 2.65 | 0.004 |
| Translation | HSD | 2.62 | 0.014 |
| Ribosome PATH ko03010 | HSD | 2.59 | 0.005 |
| Pyrimidine metabolism PATH ko00240 | HSD | 2.54 | 0.004 |
| Nucleotide metabolism | HSD | 2.52 | 0.029 |
| Amino sugar and nucleotide sugar metabolism PATH ko00520 | HSD | 2.46 | 0.010 |
| Bacterial secretion system PATH ko03070 | HSD | 2.42 | 0.000 |
| Homologous recombination PATH ko03440 | HSD | 2.22 | 0.006 |
| Folding sorting and degradation | HSD | 2.16 | 0.003 |
| Histidine metabolism PATH ko00340 | HSD | 2.10 | 0.001 |
| Biofilm formation Vibrio cholerae PATH ko05111 | HSD | 2.10 | 0.011 |
| Xenobiotics biodegradation and metabolism | HSD | 2.09 | 0.015 |
| Mismatch repair PATH ko03430 | HSD | 2.02 | 0.007 |
| DNA replication PATH ko03030 | HSD | 2.02 | 0.007 |
| Membrane transport | B | 3.22 | 0.007 |
| ABC transporters PATH ko02010 | B | 3.15 | 0.024 |
| Phosphotransferase system PTS PATH ko02060 | B | 2.75 | 0.001 |
| Quorum sensing PATH ko02024 | B | 2.68 | 0.008 |
| Metabolism of cofactors and vitamins | B | 2.64 | 0.007 |
| Fructose and mannose metabolism PATH ko00051 | B | 2.57 | 0.000 |
| Cellular community prokaryotes | B | 2.47 | 0.049 |
| Porphyrin and chlorophyll metabolism PATH ko00860 | B | 2.46 | 0.014 |
| Folate biosynthesis PATH ko00790 | B | 2.35 | 0.000 |
| Pentose phosphate pathway PATH_ko00030 | B | 2.29 | 0.002 |
| Arginine biosynthesis PATH ko00220 | B | 2.26 | 0.000 |
| Methane metabolism PATH ko00680 | B | 2.25 | 0.003 |
| Propanoate metabolism PATH ko00640 | B | 2.12 | 0.029 |
| Lysine biosynthesis PATH ko00300 | B | 2.11 | 0.001 |
| Valine leucine and isoleucine biosynthesis PATH ko00290 | B | 2.10 | 0.011 |
| Selenocompound metabolism PATH ko00450 | B | 2.00 | 0.001 |

LEfSe analyses displaying predicted enrichment (p ≤0.05, LDA ≥ 2) of functional pathways based on PICRUSt2 predicted KEGG Orthologs between the diets. The p values were derived from Wilcoxon signed-rank tests.

B, Baseline; LSD, low spice diet; MSD, moderate spice diet; HSD, high spice diet

## Supplementary Table 8: Predicted enrichment of functional genes based on KEGG Orthologs following each diet compared to baseline in participants at risk of CVD.

| **Comparison** | **Diet** | **LDA Score** | **P Value** |
| --- | --- | --- | --- |
| ***LSD vs. Baseline*** |  |  |  |
| K10439 | B | 2.07 | 0.038 |
| K02025 | B | 2.03 | 0.038 |
| ***MSD vs. Baseline*** |  |  |  |
| K10119 | B | 2.18 | 0.016 |
| K10117 | B | 2.18 | 0.028 |
| K10118 | B | 2.11 | 0.025 |
| K10439 | B | 2.11 | 0.004 |
| K07496 | B | 2.08 | 0.015 |
| K02529 | B | 2.04 | 0.023 |
| K07720 | B | 2.02 | 0.045 |
| ***HSD vs. Baseline*** |  |  |  |
| K03205 | HSD | 2.23 | 0.003 |
| K03406 | HSD | 2.19 | 0.014 |
| K10117 | B | 2.36 | 0.001 |
| K10119 | B | 2.35 | 0.001 |
| K10118 | B | 2.28 | 0.002 |
| K10439 | B | 2.27 | 0.000 |
| K07496 | B | 2.24 | 0.000 |
| K07718 | B | 2.19 | 0.002 |
| K07720 | B | 2.16 | 0.002 |
| K02529 | B | 2.14 | 0.001 |
| K10440 | B | 2.06 | 0.000 |
| K07816 | B | 2.01 | 0.003 |

LEfSe analyses displaying predicted enrichment (p ≤ 0.05, LDA ≥ 2) of functional genes based on KEGG Orthologs regrouped into KEGG pathways. The p values were derived from Wilcoxon signed-rank tests.

B, Baseline; LSD, low spice diet; MSD, moderate spice diet; HSD, high spice diet

# Supplementary Figure 1: CONSORT flow diagram.


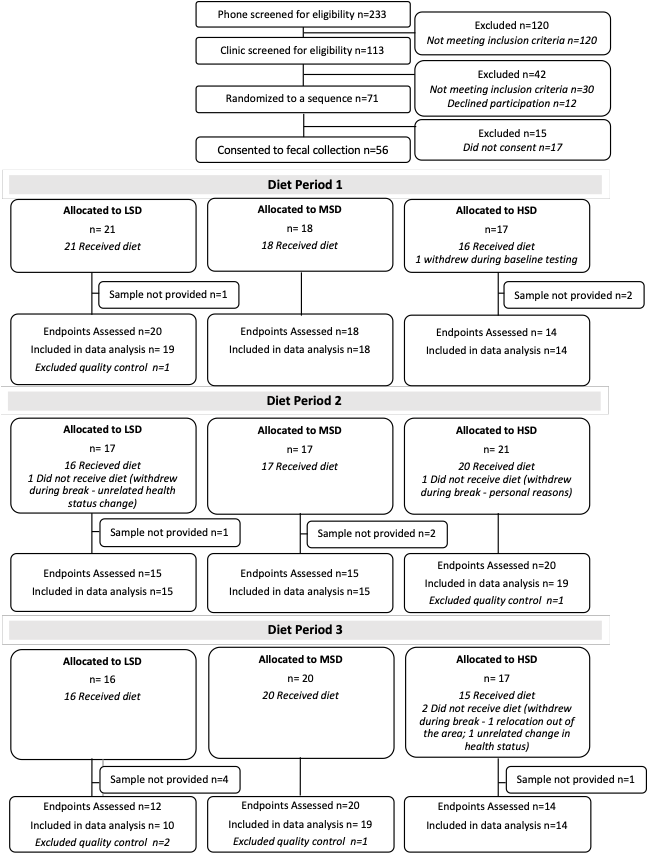


LSD, low spice diet; MSD, moderate spice diet; HSD, high spice diet
